# Computational trans-omics approach characterised methylomic and transcriptomic involvements and identified novel therapeutic targets for chemoresistance in gastrointestinal cancer stem cells

**DOI:** 10.1038/s41598-018-19284-3

**Published:** 2018-01-17

**Authors:** Masamitsu Konno, Hidetoshi Matsui, Jun Koseki, Ayumu Asai, Yoshihiro Kano, Koichi Kawamoto, Naohiro Nishida, Daisuke Sakai, Toshihiro Kudo, Taroh Satoh, Yuichiro Doki, Masaki Mori, Hideshi Ishii

**Affiliations:** 1 Department of Frontier Science for Cancer and Chemotherapy, Osaka University, Osaka, 565-0871 Japan; 2 Department of Medical Data Science, Graduate School of Medicine, Osaka University, Osaka, 565-0871 Japan; 3 Faculty of Data Science, Shiga University, Shiga, 522-8522 Japan; 4 Department of Gastroenterological Surgery, Graduate School of Medicine, Osaka University, Osaka, 565-0871 Japan

## Abstract

We investigated the relationship between methylomic [5-methylation on deoxycytosine to form 5-methylcytosine (5mC)] and transcriptomic information in response to chemotherapeutic 5-fluorouracil (5-FU) exposure and cisplatin (CDDP) administration using the ornithine decarboxylase (ODC) degron-positive cancer stem cell model of gastrointestinal tumour. The quantification of 5mC methylation revealed various alterations in the size distribution and intensity of genomic loci for each patient. To summarise these alterations, we transformed all large volume data into a smooth function and treated the area as a representative value of 5mC methylation. The present computational approach made the methylomic data more accessible to each transcriptional unit and allowed to identify candidate genes, including the tumour necrosis factor receptor-associated factor 4 (*TRAF4*), as novel therapeutic targets with a strong response to anti-tumour agents, such as 5-FU and CDDP, and whose significance has been confirmed in a mouse model *in vivo*. The present study showed that 5mC methylation levels are inversely correlated with gene expression in a chemotherapy-resistant stem cell model of gastrointestinal cancer. This mathematical method can be used to simultaneously quantify and identify chemoresistant potential targets in gastrointestinal cancer stem cells.

## Introduction

The methylation of deoxycytosine to form 5-methylcytosine (5mC) is one of the most important features of cancer^1–4^ which dynamically changes during carcinogenesis, metastasis and tumour reccurrence^5^. Therefore, investigating the relationship between DNA methylation and transcription is important for the interpretation of cellular responses and development of novel therapeutic strategies. Extensive DNA methylation and transcription analyses have provided large quantities of data, and it is difficult to identify critical genes related to cancer development from these data. We expressed DNA methylation profiles as smooth functions using Gaussian functions to extract appropriate information from the data. Tumours contain a subpopulation of cells, called cancer stem cells (CSCs), which are self-renewing and tumorigenic and play a role in the resistance against chemotherapy and radiotherapy^6–8^; therefore, we aimed to determine the efficient methods of identifying therapeutic targets using a CSC model of ornithine decarboxylase (ODC)^3–5^ to characterize intracellular events based on the 5mC methylome and transcriptome data. ODC is reportedly an important enzyme for the maintenance and chemoresistance of CSCs^9–12^. In this study, we used the ODC system as a CSC model to establish a new trans-omics model for DNA methylation and transcription. We identified several candidates, including the tumour necrosis factor receptor-associated factor 4 (*TRAF4*), as candidates for conferring resistance to anti-cancer drugs in CSCs of gastrointestinal cancer.

## Results

### Standard analysis of gene expression and DNA methylation

**Figure 1:**
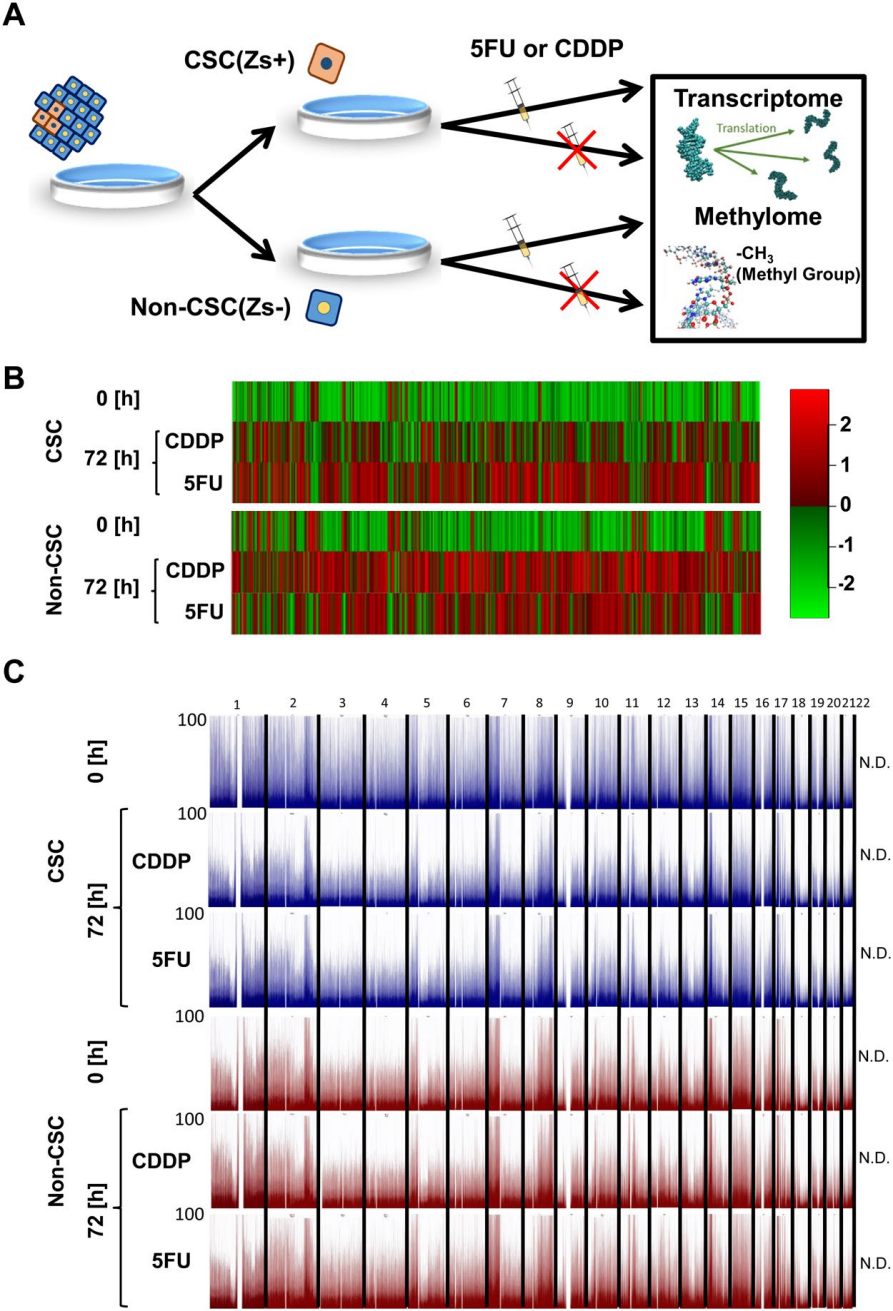
Global methylation and expression level analysis of CSCs and non-CSCs. (**A**) Experimental scheme of global transcriptome and methylome analysis. Zs Green expressing CSCs (Zs+) and low expressing non-CSCs (Zs−) were separated by cell sorting and cultured in the presence or absence of anti-cancer drugs for 72 hrs. (**B**) Heatmap for global transcriptome analysis. (**C**) Manhattan plot for global methylome analysis. Vertical axis represents the depth of sequence data.

To determine the differences between drug responses in Zs+ CSCs and Zs− non-CSCs, we exposed cells to 5-FU or CDDP for 48 h. We performed an extensive analysis of gene expression and DNA methylation (Fig. 1A). The expression of most genes was upregulated after anti-cancer drug treatment (Fig. 1B). Moreover, DNA methylation decreased in all autosomal chromosomes in Zs+ CSCs, but not in Zs− CSCs, after anti-cancer drug treatment (Fig. 1C). These data suggested a relationship between gene expression and DNA methylation levels. In addition, our findings showed that gene expression and DNA methylation are altered in Zs+ CSCs after treatment with anti-cancer drugs. Because CSCs are drug resistant, we performed gene set enrichment analysis using microarray data to identify the genes which contribute to drug resistance. Although we attempted to identify gene sets which were enriched in Zs+ CSCs but not in Zs− non-CSCs after 5FU or CDDP treatment, the background noise, which may be due to the complexity of cancer cell populations and molecular regulations at trans-omic levels such as methylation and transcription, attenuated the identification of any gene set which was responsible for the nature of CSCs. On treatment with anti-cancer drugs, the level of DNA methylation was altered in chemoresistant CSCs, but not in non-CSCs. Therefore, in this study, we aimed to simultaneously analyse the transcriptome and methylome data to identify the genes responsible for drug resistance.

### New trans-omics analysis of transcription and DNA methylation

We first summarised the information on 5mC methylation from the data as follows:

Consider the observations for methylation level $$\{{x}_{ij\alpha };\,i=1,\ldots ,\,n,\,\,j=1,\ldots ,\,p,\,\alpha =1,\ldots ,\,{n}_{ij}\}$$, where $${x}_{ij\alpha }$$ is a value of the methylation level for the *i*th observation and *j*th gene at *α*th genetic loci. Data on the loci are presented as intervals. It is logical to assume that each methylation level $${x}_{ij\alpha }$$ is a discretised realisation of function $${x}_{ij}(l)$$ at locus $${l}_{ij\alpha }$$; therefore, we transformed the observed methylation levels $$\{{x}_{ij\alpha };\,\alpha =1,\ldots ,\,{n}_{ij}\}$$ into functions $${x}_{ij}(l)$$$$.$$ Furthermore, we assumed that the methylation level peaked at the centre of each interval of methylated loci and gradually decreased as it moved away from the centre. One of the most effective techniques for transforming the data into a function is the basis expansion method^13^. Using this idea, we assume that the function $${x}_{ij}(l)$$ is expressed as the following linear combination:

1 $${x}_{ij}(l)=\sum _{\alpha =1}^{{n}_{ij}}{x}_{ij\alpha }\,{\varphi }_{ij\alpha }(l),$$

where ${\varphi }_{ij\alpha }(l)$ is a probability density function of Gaussian distribution:

2 $${\varphi }_{ij\alpha }(l)=\frac{1}{\sqrt{2\pi {s}_{ij\alpha }^{2}}}\exp \{-\frac{{(l-{l}_{ij\alpha })}^{2}}{2{s}_{ij\alpha }^{2}}\},$$

where ${s}_{ij\alpha }^{2}$ indicates that the methylation interval for ${x}_{ij\alpha }$ equals $6{s}_{ij\alpha }$. These functions are constructed so that peak positions of ${\varphi }_{ij\alpha }(l)$ coincide with the loci ${l}_{ij\alpha }$. Then, Equation (1) was constructed so that ${x}_{ij\alpha }$ equals the integration of each term over the whole region. We can treat the methylation level at the continuous loci rather than discrete ones by smoothing the observed data.

**Figure 2:**
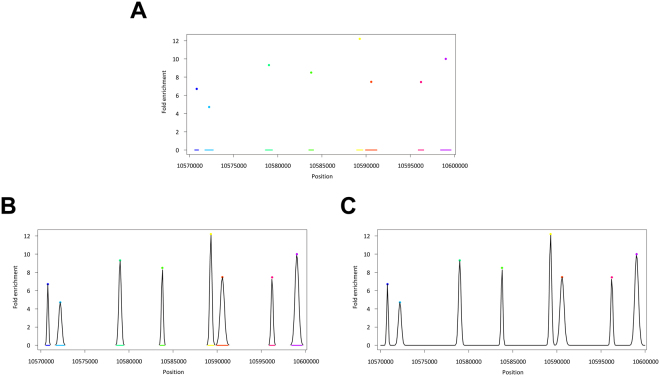
Procedure for calculating the area of methylation. (**A**–**C**) Illustration for transforming the methylation level data into a function. (**A**) Example of observed methylation levels (points) and methylation regions (segments below). (**B**) Fitted Gaussian functions (black curves) for each methylation levels. (**C**) By summing up these functions, we obtained a methylation function.

An example of a curve ${x}_{ij}(l)$ for methylation levels is presented in Fig. 2A–C. We obtained total methylation levels at arbitral loci by integrating (1) over [*a*, *b*], where *a* and *b* are arbitral genetic loci. For example, a total methylation level in the promoter region is obtained by setting $a=-\infty$ and *b* as the endpoint of the promoter region. This integration corresponds to the calculation of cumulative distribution of Gaussian distribution, so it is difficult to analytically calculate if $-\infty  < a < b < \infty$; however, it can be numerically calculated using standard software. We used this value for the trans-omics analysis to summarise the methylation data.

**Figure 3:**
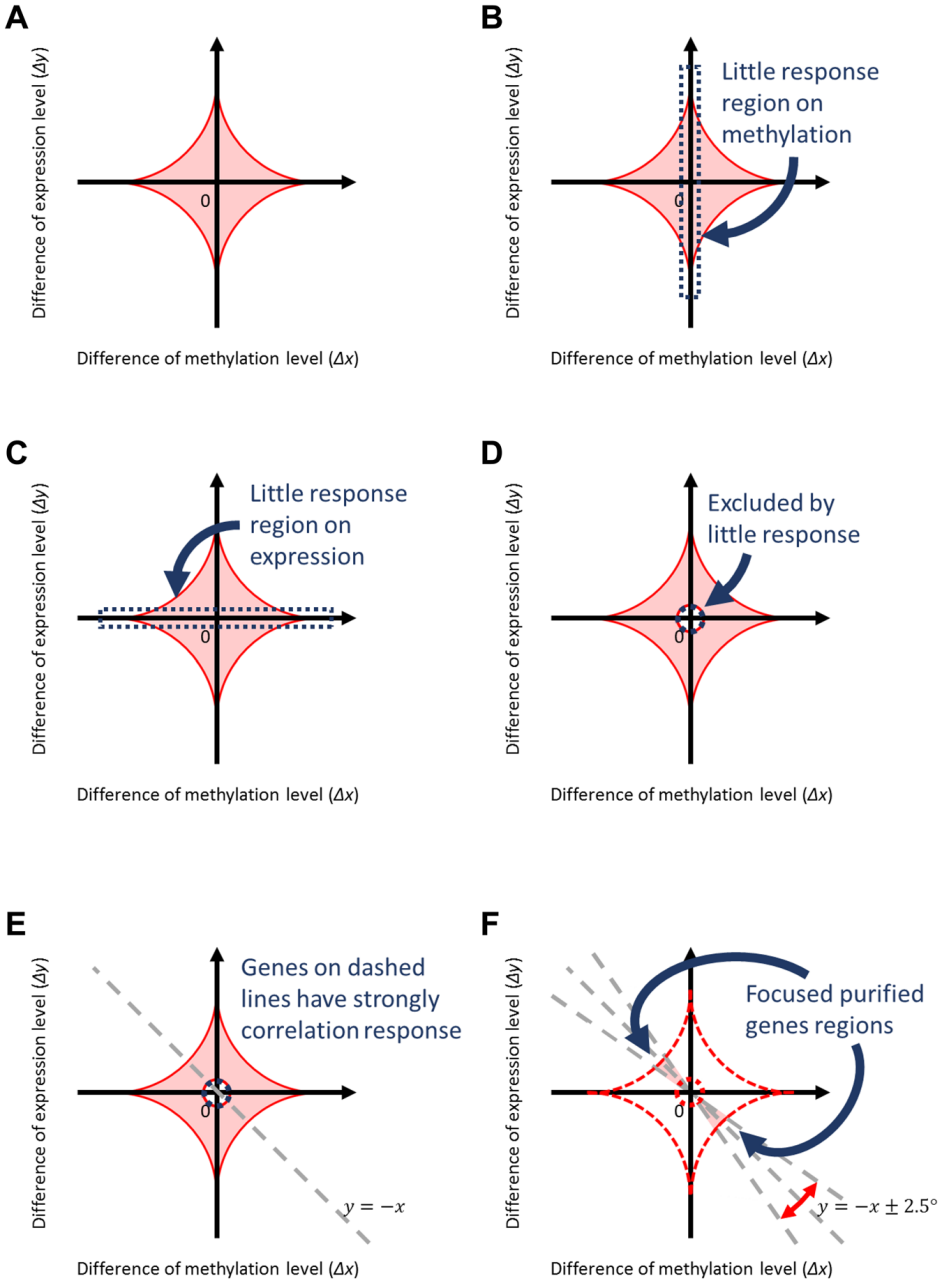
Illustration of the procedure for extracting genes. Genes with altered methylation or expression after anti-cancer drug treatment were identified.

**Figure 4:**
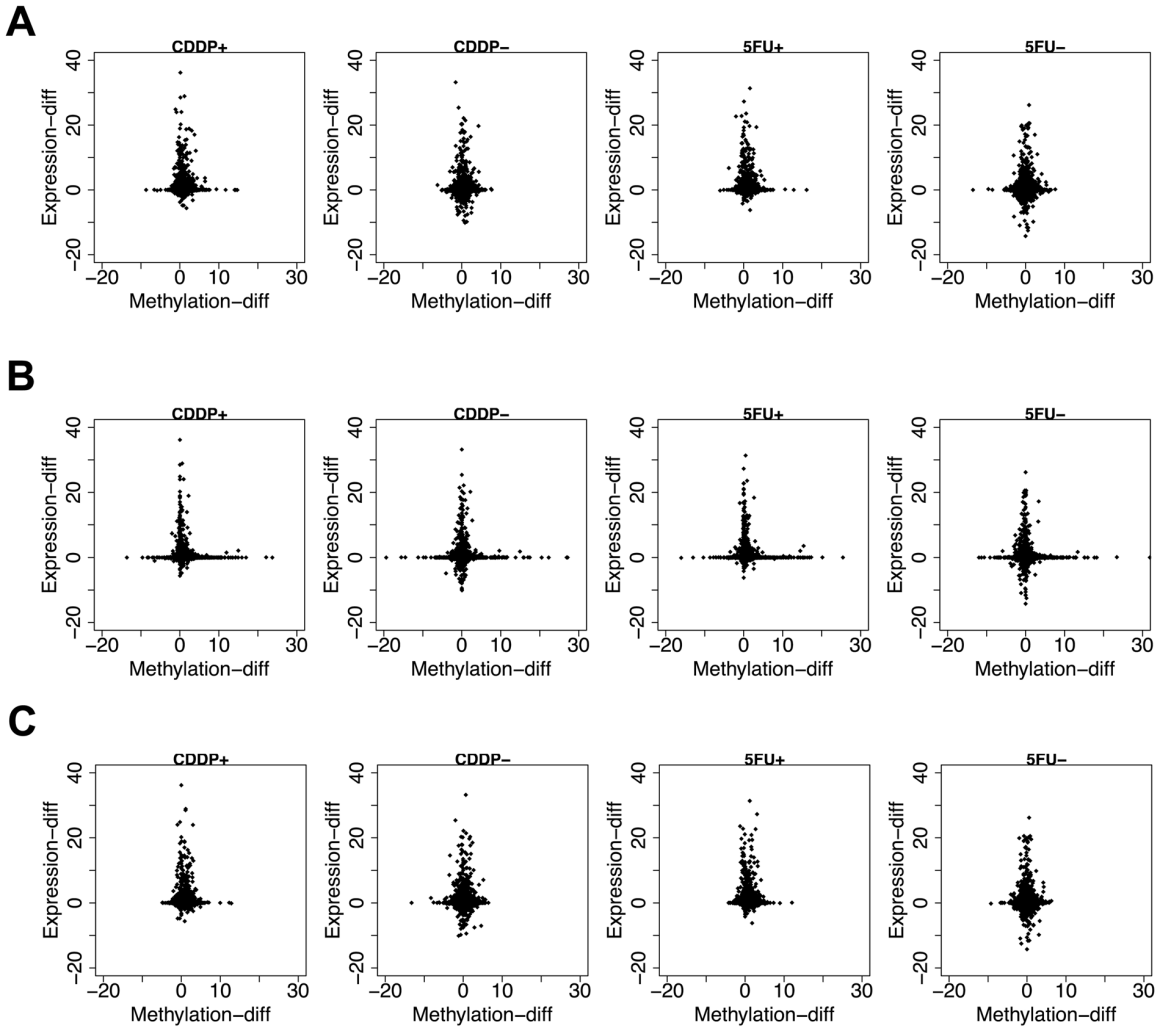
Relationship between methylation and expression levels. (**A**–**C**) Show differences in methylation and expression levels between the exposure time and 72 h after treatment. X-axis indicates the methylation area calculated by integrating the function depicted in Fig. 2C. Y-axis represents the differences in expression levels. (**A**) Upstream of genes. (**B**) Inside genes. (**C**) Downstream of genes.

**Figure 5:**
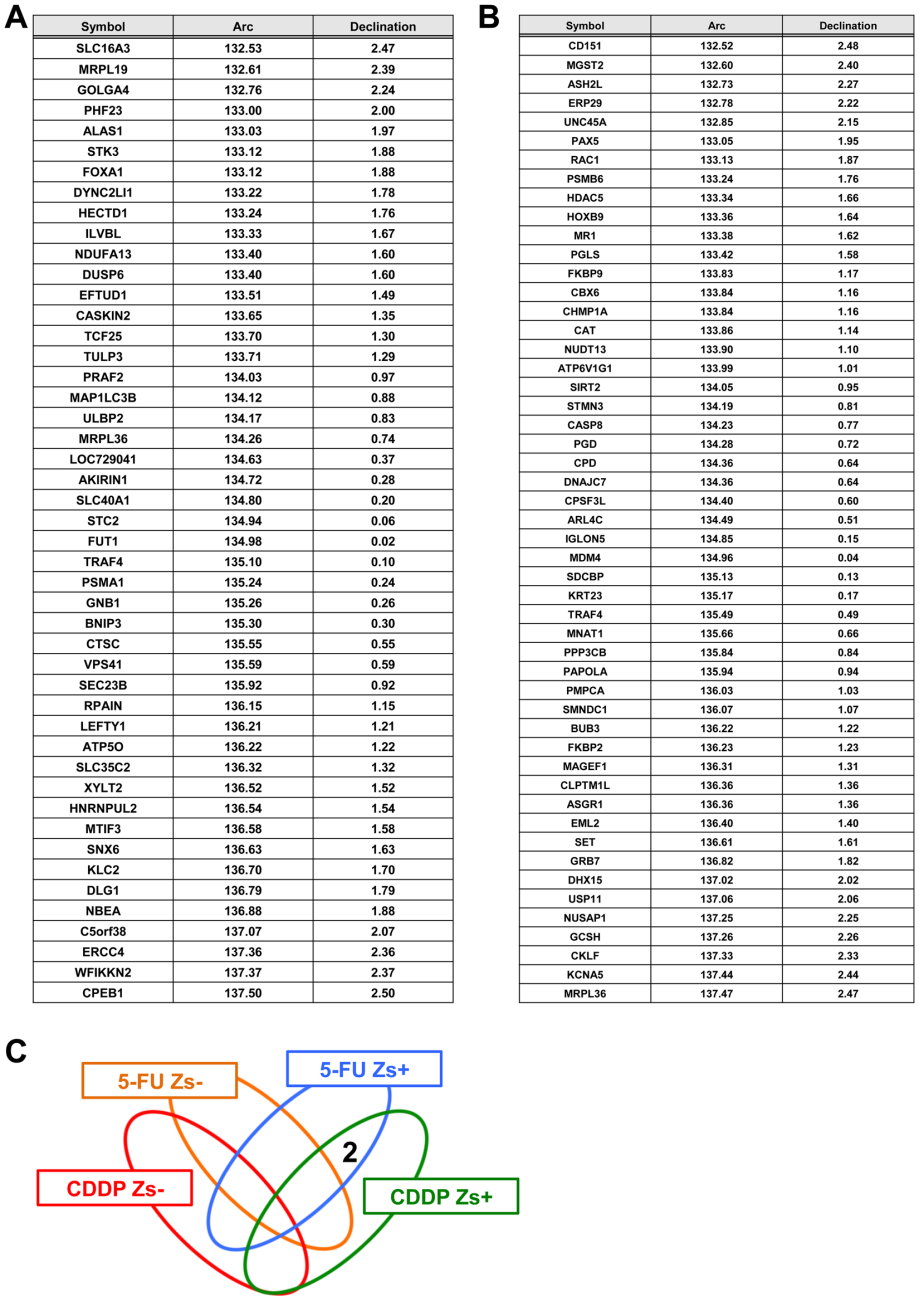
Identification of genes correlated with the expression and methylation of CSCs. Identification of genes in the “±2.5°” range from “y = −x”. The angles of polar representation for each gene are shown in the Arc column. The values in the declination column show each deviation from the angle of −45° or 135° in polar coordinate. Methylation was upstream of the gene. (**A**) The genes in the second quadrant of CSCs treated with 5-FU for 72 hrs. (**B**) The genes in the second quadrant of CSCs treated with CDDP for 72 hrs. (**C**) Venn diagram of genes in the “±2.5°” range from “y = −x”. There were three common genes between CSCs treated with 5-FU and CDDP.

Next, we calculated the differences in methylation and expression between the onset of anti-cancer drug exposure and 72 h after treatment. We focused on genes that affected methylation and expression and screened them as follows:

We denote ${\rm{\Delta }}{x}_{ij}$ and ${\rm{\Delta }}{y}_{ij}$ as differences in methylation and expression between the two time points for the *i*th subject and *j*th gene, respectively. We plotted the values of methylation and expression levels as shown in Fig. 3A. These values were standardised so that the average was 0 and standard deviation was 1. Small absolute values $(|{\rm{\Delta }}{x}_{ij}|\approx 0,\,|{\rm{\Delta }}{y}_{ij}|\approx 0)$ indicated little effect of anti-cancer drugs on methylation and expression, respectively, for *j*th gene (Fig. 3B,C). We calculated the squared norm ${r}_{ij}^{2}={\rm{\Delta }}{x}_{ij}^{2}+{\rm{\Delta }}{y}_{ij}^{2}$, and then excluded 5% of genes with smaller ${r}_{ij}^{2}$ (Fig. 3D). In our plots, the genes with the strongest response to anti-cancer drugs were plotted around the line “$y=\pm x$” (Fig. 3E). Higher methylation and lower gene expression were indicated in the zone around the line “$y=-x$.” On liberation from the negative correlation line, there was not only a weak correlation but also large margin of error. Therefore, in this study, the group of genes in the “$\pm 2.5^\circ$” range from “$y=-x$” was selected for further analysis (Fig. 3F). We identified specific genes that were present in the CDDP + and 5FU+ groups but not in CDDP− and 5FU− groups. To identify the critical genes that correlated with expression and methylation after treatment with anti-cancer drugs, we identified genes in the “ ± 2.5°” range from “y = −x” (Fig. 4A–C, Supplemental Tables 1–11, Fig. 5A,B). DNA methylation upstream of the gene is related to gene expression. Therefore, we identified genes using DNA methylation data upstream of the gene. We successfully identified two genes that were enriched in Zs+ CSCs treated with 5FU or CDDP, but not in Zs− non-CSCs treated with 5FU or CDDP (Fig. 5C). Housekeeping genes, such as GAPDH and β-actin, were used as controls. We observed no changes in their expression before and after anti-cancer drug treatment (Supplemental Table 12).

Although we used “±2.5°” as the strong correlation range, it may not always be the best in all cases. It should be customised for each system. In this study, the range was spread from “0°” in small increments until we could identify the specific genes which were present in the high correlation region, i.e. CDDP and 5FU, for CSCs and not present in the region for non-CSCs.

### Confirmation of critical targets using an animal model

**Figure 6:**
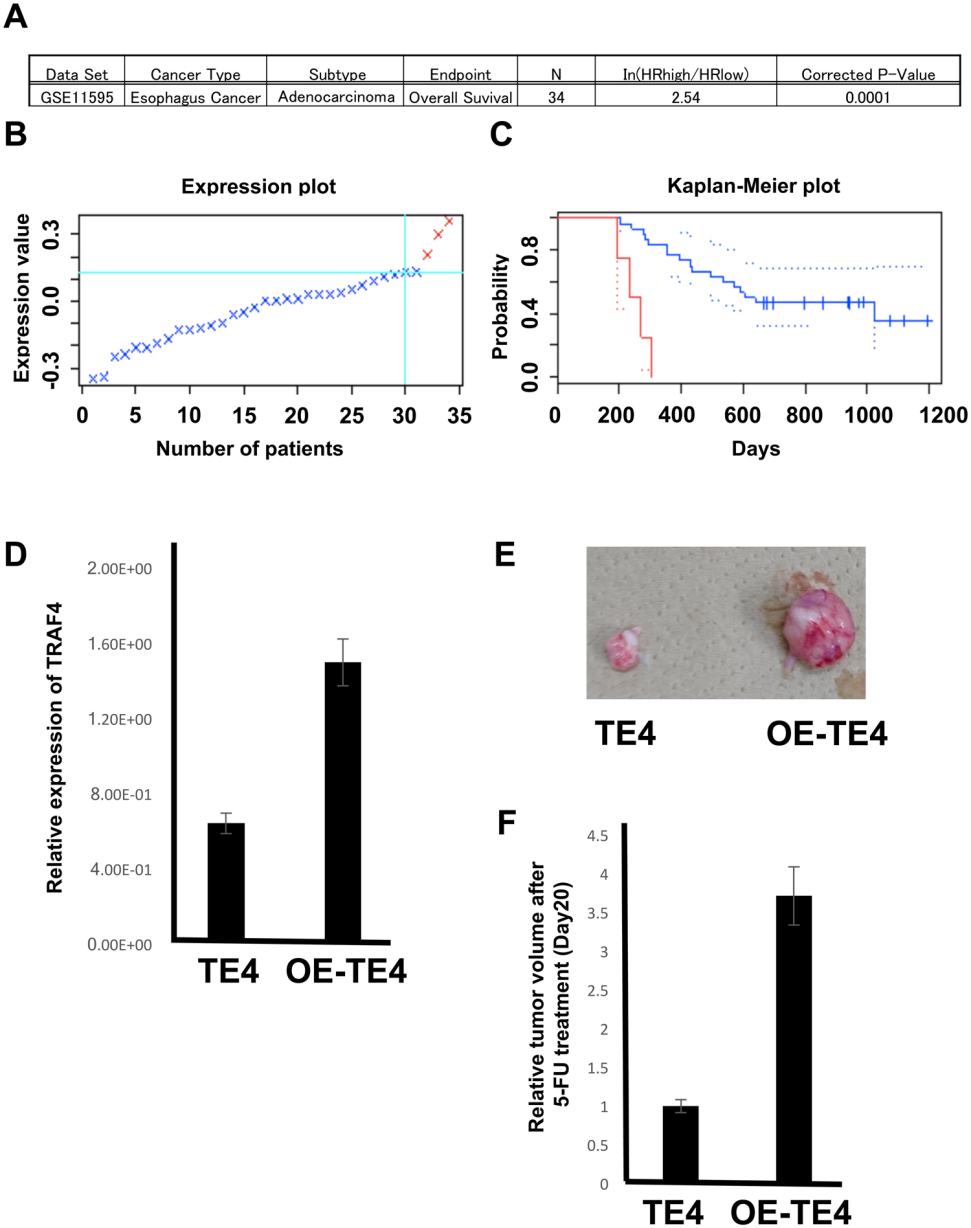
PrognoScan analysis of *TRAF4* in oesophageal cancer. (**A**) Oesophageal cancer data posted in PrognoScan. (**B**) *TRAF4* expression plot. Red plots indicate patients with highly expressed *TRAF4*. Blue plots indicate patients with low *TRAF4* expression. (**C**) Kaplan–Meier plot. Red line indicates patients with high *TRAF4* expression. Blue line indicates patients with low *TRAF4* expression. (**D**) Quantitative RT-PCR of *TRAF4* expression level in the parental TE4 and *TRAF4*-overexpressing OE-TE4 cells. The data were normalised by GAPDH expression level. (**E**) Representative tumour tissues excised from mice 20 days after tumour volumes reached 100 mm^3^, and 5-FU (20 mg/kg) was administered every 2 days. (**F**) Relative tumour volumes are shown 20 days after tumour volumes reached 100 mm^3^, and 5-FU was administered, corresponding to (**E**).

To investigate the relationship between the expression of these two genes and overall survival, we analysed the expression of these genes in oesophageal cancer using the gene expression omnibus database GSE11595^14^ and PrognoScan. *MRPL36* was not related to the prognosis of oesophageal cancer (Supplemental Fig. 1). However, *TRAF4* might be a critical gene for drug resistance. PrognoScan analysis revealed that higher *TRAF4* expression was associated with bad prognosis in all gene sets (Fig. 6A–C). To determine whether *TRAF4* is a critical gene for drug resistance, we overexpressed *TRAF4* (OE-TE4 cells) and confirmed that the expression level of *TRAF4* in OE-TE4 was 2.5-fold higher than that in parental TE4 cells (Fig. 6D). We then inoculated the OE-TE4 cells subcutaneously in immune-deficient mice. Both parental TE4 and OE-TE4 tumour volumes reached 100 mm^3^ in 10 days, which showed no significant differences in the durations of tumour development (data not shown). After the tumour volume reached to 100 mm^3^, the mice were administered 5-FU (20 mg/kg) every 2 days; the observation of mice for 20 days indicated that the tumour volume of parental TE4, but not OE-TE4, was significantly suppressed by 5-FU treatment compared with controls (Fig. 6E,F), indicating that *TRAF4* plays a role in tumour development when exposed to 5-FU, and that *TRAF4* may be a critical target for overcoming chemotherapeutic resistance of CSCs. This study demonstrated that this novel trans-omics approach for analysing transcription and DNA methylation can identify genes which are critical for drug resistance.

## Discussion

In this study, we investigated the profiles of 5mC DNA methylation using smooth functions to extract the underlying information. The areas of these functions were used as the levels of methylation. In our Gaussian fitting approach, we defined s__ijα_ so that the value when multiplied by 6 equals the methylation interval for x__ijα_, because the Gaussian type function generally includes 99.73% of their distribution within ±3σ (σ: standard deviation) from the centre of the peak. Furthermore, the differences in methylation and expression levels were compared between the time of exposure to anti-cancer drugs and 72 h after treatment to detect genes with altered levels of methylation and expression. As previously mentioned, the area of |∆x__ij_ | or |∆y__ij_ | close to zero shows little effect of anti-cancer drugs on methylation and expression, respectively. The genes with a strong correlation between methylation and expression levels are concentrated around the line “y = −x”. Therefore, we could find some genes that lead to a decrease in methylation level and increase in expression, or that show an increase in methylation and decrease in expression. However, as shown in Fig. 4, most genes had little correlation between the changes in methylation and expression after exposure to anti-tumour agents. In addition, it is important that the method be established to identify genes having a strong correlation between methylation an dexpression.

This analysis allowed the identification of *TRAF4* as an important gene for chemoresistance. *TRAF4*, a member of TRAF family^15–17^, was expressed in breast carcinomas and was the first TRAF member to be upregulated in human carcinomas^18^. Abnormal *TRAF4* expression has been reported in certain cancers, including breast, lung and prostate cancers^19,20^. *TRAF4* is expressed in the nucleus and is correlated with poor prognosis in breast cancer patients^21^. Moreover, *TRAF4* expression was associated with invasion, migration and metastasis in breast cancer. *TRAF4* is regulated by TGF-β signaling^22^ and is highly expressed in lung cancer. It may be a possible molecular target for lung cancer therapy^23^. The involvement of *TRAF4* in the biological behaviour of cancer cells has been reported. However, to the best of our knowledge, this study showed for the first time that *TRAF4* is important for the possible regulation of functions in CSCs of human oesophageal cancer, which was indicated by the combination of computational and animal studies, further supporting the rationale for the large scale screening of therapeutic targets of CSC drug development.

## Methods

### Cell culture and sorting

We purchased the human gastrointestinal cancer cell line of the oesophagus TE-4 from the Japanese Collection of Research Bioresources Cell Bank (Ibaraki, Japan). TE-4 cells were cultured in Dulbecco’s modified Eagle’s medium (DMEM; Sigma-Aldrich, St. Louis, MO, USA) supplemented with 10% foetal bovine serum (FBS; Hyclone, Logan, UT, USA) and penicillin–streptomycin (Sigma-Aldrich) at 37 °C in 5% CO_2_. Retroviruses were prepared using the platinum-A retroviral packaging cell line (Plat-A). Plat-A cells were cultured in DMEM supplemented with 10% FBS, 100 U/ml penicillin (Life Technologies, Gaithersburg, MD, USA), 1 µg/ml puromycin (Sigma-Aldrich) and 10 µg/ml blasticidin (Sigma-Aldrich). To generate retroviruses, we transfected Plat-A cells with the retroviral vector pQCXIN-ZsGreen-cODC, which encodes the ZsGreen-cODC fluorescent fusion protein using FuGENE6 transfection reagent (Promega Corp., Madison, WI, USA). The medium was changed 1 day after transfection, and 1 day later, the supernatant containing the retroviruses was collected. To induce cancer cell formation, we added this supernatant and 6 mg/ml polybrene (Sigma-Aldrich) to DMEM containing the cultured cancer cells. The cells with high ZsGreen-cODC (Zs+) and low ZsGreen-cODC (Zs−) expressions were separated after two rounds of fluorescence-activated cell sorting (FACS) and defined as CSCs or differentiated cancer cells, respectively. Cells were washed with phosphate-buffered saline and trypsinised using 0.25% trypsin-ethylenediaminetetraacetic acid (Life Technologies). Then, cells were sorted using a BD FACS Aria II cell sorting system (Becton-Dickinson, Franklin Lakes, NJ, USA), after which CSCs and non-CSCs were cultured in the presence of 5-FU (10 μM) or CDDP (10 μM) for 72 h.

### DNA methylation analysis

DNA methylation was analysed in CSCs and non-CSCs. Methylated proteins were immunoprecipitated from the cell lysate using a methylation-binding protein. Samples were sequenced (Takara, Kyoto, Japan) to obtain whole genome-wide DNA methylation data.

### Microarray analysis

The extracted total RNA (500 ng) was labelled with cyanine-3 using the low input quick amp labelling kit (Agilent Technologies, Tokyo, Japan) after checking for sufficient quality of microRNA microarray experiments. The cRNA yield and dye incorporation were monitored using a Nanodrop ND-2000 spectrophotometer. Labelled RNAs were hybridised to the Agilent mouse GE 8 × 60 K microarray in a rotating Agilent hybridisation oven for 17 h at 65 °C. After hybridization, the microarrays were washed at room temperature for 1 min with GE wash buffer 1 (Agilent) and then with GE wash buffer 2 (Agilent Technologies) at 37 °C. Microarrays were then dried and briefly centrifuged. Fluorescence signals were determined using an Agilent DNA microarray scanner (G2565CA) after stringent washes with GE wash buffers 1 and 2 (Agilent Technologies) for 1 min each. The fluorescence signals were analysed using feature extraction software 10.10 (Agilent Technologies).

### PrognoScan analysis

Relationships between gene expression levels and cancer prognosis were analysed using the PrognoScan data base (http://www.abren.net/PrognoScan/). Use of these data does not require written informed consent because they are available online.

### Animal experiments

Parental TE4 cells or KD-TE4 cells were injected subcutaneously in 4–6-week-old female NOD-SCID mice with diabetes/severe combined immunodeficiency (CLEA Japan, Tokyo, Japan). After the tumour volume reached 100 mm^3^, the mice were administered 5-FU (20 mg/kg) every 2 days. The tumours were resected 20 days after the tumour volume reached 100 mm^3^. All methods were carried out in accordance with relevant guidelines and regulations, under the experimental protocol, which was approved by the licensing committee of animal experiment at Osaka University.

## Electronic supplementary material

Supplementary Data
