# Supplementary material for: Computational trans-omics approach characterised methylomic and transcriptomic involvements and identified novel therapeutic targets for chemoresistance in gastrointestinal cancer stem cells
Source: Sci Rep. 2018 Jan 17;8:899. doi: 10.1038/s41598-018-19284-3 (PMC5772492; doi:10.1038/s41598-018-19284-3)
Supplement: Supplementary file 1 — Supplementary Data [file 41598_2018_19284_MOESM1_ESM.doc]

Computational trans-omics approach characterised methylomic and transcriptomic involvements and identified novel therapeutic targets for chemoresistance in gastrointestinal cancer stem cells

Masamitsu Konno^1,2,^*, Hidetoshi Matsui^3,^*, Jun Koseki^2,^*, Ayumu Asai^1,2^, Yoshihiro Kano^1,4^, Koichi Kawamoto^4^, Naohiro Nishida^4^, Daisuke Sakai^1^, Toshihiro Kudo^1^, Taroh Satoh^1^, Yuichiro Doki^4,#^, Masaki Mori^4,#^, Hideshi Ishii^1,2,#^

^1^Department of Frontier Science for Cancer and Chemotherapy; ^2^Department of Medical Data Science Graduate School of Medicine, Osaka University, Osaka, 565-0871 Japan; ^3^Faculty of Data Science, Shiga University, Shiga, 522-8522, Japan; ^4^Department of Gastroenterological Surgery, Graduate School of Medicine, Osaka University, Osaka, 565-0871 Japan

*These authors contributed equally to this work.

^#^To whom correspondence should be addressed:

Departments of Medical Data Science and Gastroenterological Surgery

Osaka University Graduate School of Medicine

Suita, Yamadaoka 2-2 Osaka 565-0871, Japan

Tel: +81-(0)6-6879-2641, 2640, 3251; Fax: +81-(0)6-6879-2639, 3259

E-mail: hishii@gesurg.med.osaka-u.ac.jp; mmori@gesurg.med.osaka-u.ac.jp

**Supplementary Figure 1 PrognoScan analysis of *MRPL36* gene in esophagus cancer.**

A. Esophagus cancer data posted in PrognoScan. B. *MRPL36* expression plot. Red plots mean *MPRL36* high expressed patients. Blue plots mean *MPRL36* low expressed patients. C. Kaplan–Meier plot. Red line means *MRPL36* high expressed patients. Blue line means *MRPL36* low expressed patients.

**Supplementary Table.1 Identification of genes in the “±2.5°” range from “y = −x.” The angles of polar representation for each gene are shown in the Arc column. The values in the Declination column show each deviation from the angle of −45° or 135° in the polar coordinates. Methylation was detected upstream of the gene. A. The genes in the 2nd quadrant of non-CSCs treated with 5-FU for 72 h. B. The genes in the 2nd quadrant of non- CSCs treated with CDDP for 72 h.**

**A**

| **Symbol** | **Arc** | **Declination** |
| --- | --- | --- |
| **ASB5** | **132.53** | **2.47** |
| **CCBL2** | **132.62** | **2.38** |
| **C19orf53** | **132.75** | **2.25** |
| **UBE2R2** | **132.84** | **2.16** |
| **IFI6** | **132.91** | **2.09** |
| **SMAD3** | **132.92** | **2.08** |
| **GABARAP** | **132.93** | **2.07** |
| **ERICH1** | **132.96** | **2.04** |
| **DHRS1** | **132.97** | **2.03** |
| **NKIRAS2** | **133.00** | **2.00** |
| **C9orf152** | **133.01** | **1.99** |
| **ACADS** | **133.09** | **1.91** |
| **WARS** | **133.14** | **1.86** |
| **PEMT** | **133.25** | **1.75** |
| **SV2B** | **133.36** | **1.64** |
| **BAIAP2L1** | **133.37** | **1.63** |
| **NPC2** | **133.38** | **1.62** |
| **AIFM2** | **133.62** | **1.38** |
| **MRPL24** | **133.62** | **1.38** |
| **FN1** | **133.64** | **1.36** |
| **OSBPL3** | **133.74** | **1.26** |
| **SMARCC1** | **133.75** | **1.25** |
| **CPA4** | **133.77** | **1.23** |
| **HMGN4** | **133.79** | **1.21** |
| **PAN3** | **133.83** | **1.17** |
| **PLA2G4A** | **133.90** | **1.10** |
| **CLINT1** | **133.95** | **1.05** |
| **RBM12** | **133.98** | **1.02** |
| **NSMCE1** | **133.99** | **1.01** |
| **JDP2** | **134.00** | **1.00** |
| **SCLY** | **134.01** | **0.99** |
| **KLF5** | **134.14** | **0.86** |
| **HECTD1** | **134.25** | **0.75** |
| **CHMP4B** | **134.27** | **0.73** |
| **TM2D1** | **134.31** | **0.69** |
| **TMEM69** | **134.38** | **0.62** |
| **MTX1** | **134.39** | **0.61** |
| **FBXO42** | **134.42** | **0.58** |
| **TWF2** | **134.43** | **0.57** |
| **ZNF330** | **134.49** | **0.51** |
| **PKD2L1** | **134.59** | **0.41** |
| **PLLP** | **134.77** | **0.23** |
| **F11R** | **134.83** | **0.17** |
| **JAK1** | **134.94** | **0.06** |
| **APOD** | **134.94** | **0.06** |
| **PRAME** | **135.07** | **0.07** |
| **GSTT1** | **135.14** | **0.14** |
| **PFDN5** | **135.16** | **0.16** |
| **SNX32** | **135.17** | **0.17** |
| **MRPL54** | **135.29** | **0.29** |
| **RXRA** | **135.29** | **0.29** |
| **SLC25A5** | **135.31** | **0.31** |
| **SLC39A11** | **135.38** | **0.38** |
| **CUL5** | **135.42** | **0.42** |
| **UCHL3** | **135.63** | **0.63** |
| **ASH2L** | **135.79** | **0.79** |
| **KCNJ11** | **135.85** | **0.85** |
| **BACH1** | **136.11** | **1.11** |
| **CARHSP1** | **136.11** | **1.11** |
| **OCIAD1** | **136.25** | **1.25** |
| **PMPCA** | **136.33** | **1.33** |
| **PRDX4** | **136.44** | **1.44** |
| **BUD31** | **136.61** | **1.61** |
| **TSPAN31** | **136.61** | **1.61** |
| **SSSCA1** | **136.87** | **1.87** |
| **PRDX1** | **136.94** | **1.94** |
| **FAM178B** | **137.05** | **2.05** |
| **CNP** | **137.12** | **2.12** |
| **SYTL1** | **137.28** | **2.28** |
| **HMGB3** | **137.29** | **2.29** |
| **LSM12** | **137.31** | **2.31** |
| **CLK3** | **137.33** | **2.33** |
| **S100A12** | **137.45** | **2.45** |
| **SUSD3** | **137.47** | **2.47** |
| **MSX1** | **137.48** | **2.48** |
| **RTKN** | **137.49** | **2.49** |
| **MRPS21** | **137.50** | **2.50** |

**B**

| **Symbol** | **Arc** | **Declination** |
| --- | --- | --- |
| **ROCK1** | **132.57** | **2.43** |
| **NSMCE4A** | **132.65** | **2.35** |
| **LEPROTL1** | **132.69** | **2.31** |
| **TSC22D3** | **132.69** | **2.31** |
| **RAB34** | **132.72** | **2.28** |
| **AP4E1** | **132.77** | **2.23** |
| **ATF6** | **132.90** | **2.10** |
| **QPRT** | **133.00** | **2.00** |
| **NTHL1** | **133.02** | **1.98** |
| **LGALS4** | **133.05** | **1.95** |
| **ALPPL2** | **133.16** | **1.84** |
| **TMEM86A** | **133.41** | **1.59** |
| **MRPL34** | **133.42** | **1.58** |
| **CBX6** | **133.44** | **1.56** |
| **LEFTY1** | **133.47** | **1.53** |
| **SLC25A4** | **133.50** | **1.50** |
| **KIR3DX1** | **133.56** | **1.44** |
| **ITM2C** | **133.63** | **1.37** |
| **SPCS3** | **133.73** | **1.27** |
| **EML2** | **133.76** | **1.24** |
| **ACOT8** | **133.78** | **1.22** |
| **HIPK4** | **133.82** | **1.18** |
| **BZW1** | **133.87** | **1.13** |
| **TOMM20** | **133.87** | **1.13** |
| **IGFBP2** | **133.99** | **1.01** |
| **B9D1** | **133.99** | **1.01** |
| **SEMA3A** | **133.99** | **1.01** |
| **ACSF2** | **134.06** | **0.94** |
| **C17orf49** | **134.08** | **0.92** |
| **TIA1** | **134.15** | **0.85** |
| **UFD1L** | **134.16** | **0.84** |
| **MXI1** | **134.16** | **0.84** |
| **SERPINE2** | **134.17** | **0.83** |
| **ZFAND5** | **134.20** | **0.80** |
| **STX10** | **134.24** | **0.76** |
| **C2orf47** | **134.25** | **0.75** |
| **PYCR1** | **134.28** | **0.72** |
| **ATG4C** | **134.30** | **0.70** |
| **SLC25A37** | **134.31** | **0.69** |
| **TPD52L1** | **134.36** | **0.64** |
| **GFOD1** | **134.37** | **0.63** |
| **FBXO34** | **134.48** | **0.52** |
| **ERO1L** | **134.53** | **0.47** |
| **GAPDH** | **134.58** | **0.42** |
| **PSAT1** | **134.73** | **0.27** |
| **PRKCH** | **134.82** | **0.18** |
| **TRIM7** | **134.96** | **0.04** |
| **UQCRC2** | **135.14** | **0.14** |
| **ST6GAL1** | **135.17** | **0.17** |
| **TSPAN3** | **135.24** | **0.24** |
| **PTPRE** | **135.25** | **0.25** |
| **TRAPPC2L** | **135.31** | **0.31** |
| **C11orf58** | **135.33** | **0.33** |
| **RPN2** | **135.36** | **0.36** |
| **KEAP1** | **135.43** | **0.43** |
| **MYO1C** | **135.45** | **0.45** |
| **ZNF461** | **135.49** | **0.49** |
| **SH3KBP1** | **135.52** | **0.52** |
| **THYN1** | **135.76** | **0.76** |
| **MYL6B** | **135.78** | **0.78** |
| **LDHB** | **135.85** | **0.85** |
| **OXA1L** | **135.85** | **0.85** |
| **NBL1** | **135.86** | **0.86** |
| **GRB7** | **135.90** | **0.90** |
| **EME2** | **135.90** | **0.90** |
| **SCN4A** | **135.90** | **0.90** |
| **IREB2** | **135.92** | **0.92** |
| **FCHO1** | **135.97** | **0.97** |
| **UBE2S** | **135.97** | **0.97** |
| **BCKDK** | **135.98** | **0.98** |
| **PDE6D** | **136.00** | **1.00** |
| **NEDD4L** | **136.07** | **1.07** |
| **SCP2** | **136.08** | **1.08** |
| **LONRF3** | **136.12** | **1.12** |
| **TAF1C** | **136.13** | **1.13** |
| **SLIT2** | **136.16** | **1.16** |
| **MYLIP** | **136.17** | **1.17** |
| **TPD52L2** | **136.19** | **1.19** |
| **ALDH3B2** | **136.23** | **1.23** |
| **RABGGTB** | **136.24** | **1.24** |
| **SLC33A1** | **136.26** | **1.26** |
| **GGCT** | **136.31** | **1.31** |
| **ROR1** | **136.34** | **1.34** |
| **NF1** | **136.36** | **1.36** |
| **SCRIB** | **136.45** | **1.45** |
| **PACSIN3** | **136.51** | **1.51** |
| **FKBP2** | **136.53** | **1.53** |
| **WDR13** | **136.54** | **1.54** |
| **TOM1** | **136.57** | **1.57** |
| **SNRNP27** | **136.58** | **1.58** |
| **PTPN9** | **136.59** | **1.59** |
| **CHKB** | **136.65** | **1.65** |
| **UBE4A** | **136.66** | **1.66** |
| **TOP2A** | **136.70** | **1.70** |
| **VCL** | **136.88** | **1.88** |
| **NUDT17** | **136.89** | **1.89** |
| **AACS** | **136.89** | **1.89** |
| **DIP2A** | **136.96** | **1.96** |
| **NSUN4** | **137.05** | **2.05** |
| **ELOVL5** | **137.06** | **2.06** |
| **SERPINB6** | **137.14** | **2.14** |
| **NKX2-4** | **137.15** | **2.15** |
| **HOXB2** | **137.29** | **2.29** |
| **FAAH2** | **137.33** | **2.33** |
| **CDA** | **137.46** | **2.46** |
| **NUPL2** | **137.50** | **2.50** |

**Supplementary Table 2 Identification of genes in the “±2.5°” range from “y = −x.” The angles of polar representation for each gene are shown in the Arc column. The values in the Declination column show each deviation from the angle of –45° or 135° in the polar coordinates. Methylation was detected upstream of the gene. A. The genes in the 4th quadrant of CSCs treated with 5-FU for 72 h. B. The genes in 4th quadrant of CSCs treated with CDDP for 72 h.**

**A**

| **Symbol** | **Arc** | **Declination** |
| --- | --- | --- |
| **POLR2E** | **–47.42** | **2.42** |
| **PKP1** | **–47.04** | **2.04** |
| **DDRGK1** | **–46.64** | **1.64** |
| **CCT4** | **–45.76** | **0.76** |
| **STARD7** | **–45.62** | **0.62** |
| **NOP2** | **–45.48** | **0.48** |
| **RHOB** | **–45.36** | **0.36** |
| **RPAP1** | **–43.68** | **1.32** |

**B**

| **Symbol** | **Arc** | **Declination** |
| --- | --- | --- |
| **CENPN** | **–46.76** | **1.76** |
| **PTRH2** | **–46.47** | **1.47** |
| **ZNF326** | **–45.95** | **0.95** |
| **ING5** | **–45.79** | **0.79** |
| **HSPH1** | **–45.08** | **0.08** |
| **DUSP12** | **–44.98** | **0.02** |
| **CALM2** | **–44.11** | **0.89** |
| **ALG3** | **–43.48** | **1.52** |
| **LEPREL1** | **–43.05** | **1.95** |

**Supplementary Table 3 Identification of genes in the “±2.5°” range from “y = −x.” The angles of polar representation for each gene are shown in the Arc column. The values in Declination column show each deviation from the angle of –45° or 135° in the polar coordinates. Methylation was detected upstream of the gene. A. The genes in the 4th quadrant of non-CSCs treated with 5-FU for 72 h. B. The genes in the 4th quadrant of non-CSCs treated with CDDP for 72 h.**

**A**

| **Symbol** | **Arc** | **Declination** |
| --- | --- | --- |
| **HMGB2** | **–47.38** | **2.38** |
| **SLC25A3** | **–46.93** | **1.93** |
| **UBAP2** | **–46.40** | **1.40** |
| **POM121C** | **–46.35** | **1.35** |
| **IDH3B** | **–46.31** | **1.31** |
| **PPIE** | **–46.21** | **1.21** |
| **MRPS2** | **–46.14** | **1.14** |
| **MED21** | **–46.10** | **1.10** |
| **LMNB2** | **–46.04** | **1.04** |
| **GCAT** | **–44.92** | **0.08** |
| **ALKBH1** | **–44.59** | **0.41** |
| **RCC1** | **–44.25** | **0.75** |
| **ZC3H14** | **–43.83** | **1.17** |
| **ATAD2** | **–43.67** | **1.33** |
| **PAK1IP1** | **–43.48** | **1.52** |
| **ABHD5** | **–43.17** | **1.83** |
| **TFDP2** | **–43.01** | **1.99** |
| **NCBP1** | **–42.51** | **2.49** |

**B**

| **Symbol** | **Arc** | **Declination** |
| --- | --- | --- |
| **NUP88** | **–47.28** | **2.28** |
| **PPP5C** | **–46.22** | **1.22** |
| **MRPL53** | **–45.20** | **0.20** |
| **POLR3D** | **–44.89** | **0.11** |
| **BLZF1** | **–44.52** | **0.48** |
| **LAD1** | **–42.83** | **2.17** |
| **DDB2** | **–42.61** | **2.39** |

**Supplementary Table 4 Identification of genes in the “±2.5°” range from “y = −x.” The angles of polar representation for each gene are shown in the Arc column. The values in the Declination column show each deviation from the angle of –45° or 135° in polar coordinate. Methylation was detected down-stream of the gene. A. The genes in the 2nd quadrant of CSCs treated with 5-FU for 72 h. B. The genes in the 2nd quadrant of CSCs treated with CDDP for 72 h.**

**A**

| **Symbol** | **Arc** | **Declination** |
| --- | --- | --- |
| **CANX** | **132.51** | **2.49** |
| **ITGB5** | **132.61** | **2.39** |
| **SPOCK2** | **132.66** | **2.34** |
| **SDR39U1** | **133.04** | **1.96** |
| **MRPS14** | **133.07** | **1.93** |
| **SLC26A10** | **133.18** | **1.82** |
| **NSMCE4A** | **133.32** | **1.68** |
| **BNIP2** | **133.57** | **1.43** |
| **CYTH2** | **133.58** | **1.42** |
| **ZCRB1** | **133.70** | **1.30** |
| **EFNA1** | **133.73** | **1.27** |
| **UBE2Q1** | **133.81** | **1.19** |
| **MUS81** | **133.84** | **1.16** |
| **BTG1** | **133.93** | **1.07** |
| **ATG7** | **134.09** | **0.91** |
| **ASB4** | **134.29** | **0.71** |
| **RASGRP4** | **134.31** | **0.69** |
| **TSPO** | **134.33** | **0.67** |
| **PITPNM1** | **134.81** | **0.19** |
| **CRYL1** | **134.90** | **0.10** |
| **DBF4** | **134.92** | **0.08** |
| **TMED4** | **135.16** | **0.16** |
| **PDZD7** | **135.34** | **0.34** |
| **OOEP** | **135.54** | **0.54** |
| **ATOX1** | **135.54** | **0.54** |
| **RAB7A** | **135.66** | **0.66** |
| **GRPEL1** | **135.66** | **0.66** |
| **MRPL51** | **135.67** | **0.67** |
| **RMND5B** | **136.11** | **1.11** |
| **EIF4G1** | **136.15** | **1.15** |
| **HSPB8** | **136.23** | **1.23** |
| **PNPLA2** | **136.30** | **1.30** |
| **B4GALT3** | **136.42** | **1.42** |
| **TIMM44** | **136.48** | **1.48** |
| **NIPA2** | **136.53** | **1.53** |
| **KRTAP12-3** | **136.55** | **1.55** |
| **EML2** | **136.76** | **1.76** |
| **MYCBP** | **136.82** | **1.82** |
| **ELP3** | **137.08** | **2.08** |
| **FAM90A1** | **137.09** | **2.09** |
| **RAE1** | **137.09** | **2.09** |
| **EIF3M** | **137.14** | **2.14** |
| **ATP1B3** | **137.15** | **2.15** |
| **MPV17** | **137.20** | **2.20** |
| **DRAP1** | **137.26** | **2.26** |
| **DOLPP1** | **137.29** | **2.29** |
| **SQRDL** | **137.47** | **2.47** |

**B**

| **Symbol** | **Arc** | **Declination** |
| --- | --- | --- |
| **S100A4** | **132.68** | **2.32** |
| **CCDC47** | **132.83** | **2.17** |
| **ST3GAL4** | **132.88** | **2.12** |
| **CLPP** | **133.15** | **1.85** |
| **MRPS14** | **133.17** | **1.83** |
| **ITGA5** | **133.30** | **1.70** |
| **PFKP** | **133.67** | **1.33** |
| **EMP2** | **133.73** | **1.27** |
| **YARS** | **133.91** | **1.09** |
| **CFL2** | **133.96** | **1.04** |
| **NANOS2** | **134.06** | **0.94** |
| **TMEM203** | **134.22** | **0.78** |
| **MRPL20** | **134.22** | **0.78** |
| **SYNGR1** | **134.58** | **0.42** |
| **CASP4** | **134.77** | **0.23** |
| **LY96** | **135.18** | **0.18** |
| **GGCT** | **135.27** | **0.27** |
| **NETO2** | **135.68** | **0.68** |
| **MBD4** | **135.84** | **0.84** |
| **ELL2** | **136.02** | **1.02** |
| **CDC42EP5** | **136.10** | **1.10** |
| **TM7SF2** | **136.12** | **1.12** |
| **KIF2C** | **136.29** | **1.29** |
| **NDUFA6** | **136.57** | **1.57** |
| **PSMA1** | **136.70** | **1.70** |
| **POFUT1** | **136.78** | **1.78** |
| **TRIM7** | **136.89** | **1.89** |
| **HBP1** | **136.95** | **1.95** |
| **FAM89A** | **137.08** | **2.08** |
| **PON2** | **137.26** | **2.26** |
| **PGD** | **137.31** | **2.31** |
| **TLR6** | **137.38** | **2.38** |
| **RAB10** | **137.43** | **2.43** |
| **GPC2** | **137.45** | **2.45** |

**Supplementary Table 5 Identification of genes in the “±2.5°” range from “y = −x.” The angles of polar representation for each gene are shown in the Arc column. The values in the Declination column show each deviation from the angle of −45° or 135° in the polar coordinates. Methylation was detected down-stream of the gene. A. The genes in the 2nd quadrant of non-CSCs treated with 5-FU for 72 h. B. The genes in the 2nd quadrant of non-CSCs treated with CDDP for 72 h.**

**A**

| **Symbol** | **Arc** | **Declination** |
| --- | --- | --- |
| **DHX15** | **132.56** | **2.44** |
| **CBLN1** | **132.62** | **2.38** |
| **SLC11A2** | **132.67** | **2.33** |
| **PEX7** | **132.73** | **2.27** |
| **SH3TC1** | **132.87** | **2.13** |
| **C20orf24** | **132.95** | **2.05** |
| **CDC42** | **132.98** | **2.02** |
| **FKBP3** | **133.14** | **1.86** |
| **COQ10B** | **133.16** | **1.84** |
| **ALAS1** | **133.33** | **1.67** |
| **LIFR** | **133.34** | **1.66** |
| **UBR2** | **133.34** | **1.66** |
| **SCFD1** | **133.36** | **1.64** |
| **WDR54** | **133.51** | **1.49** |
| **DOT1L** | **133.57** | **1.43** |
| **TSPO** | **133.63** | **1.37** |
| **MTHFD2** | **133.71** | **1.29** |
| **RNF144A** | **133.81** | **1.19** |
| **ASCC1** | **133.84** | **1.16** |
| **HADH** | **133.91** | **1.09** |
| **PPAN** | **133.96** | **1.04** |
| **BLVRA** | **133.99** | **1.01** |
| **WDR48** | **134.03** | **0.97** |
| **TM6SF1** | **134.07** | **0.93** |
| **SRP54** | **134.18** | **0.82** |
| **DCTN4** | **134.18** | **0.82** |
| **RHOD** | **134.21** | **0.79** |
| **BCL7B** | **134.21** | **0.79** |
| **CTSZ** | **134.24** | **0.76** |
| **PSMG3** | **134.32** | **0.68** |
| **SLC26A10** | **134.37** | **0.63** |
| **STRA6** | **134.39** | **0.61** |
| **HSCB** | **134.51** | **0.49** |
| **FAM50A** | **134.69** | **0.31** |
| **FXYD2** | **134.97** | **0.03** |
| **HERPUD2** | **135.21** | **0.21** |
| **CLDND1** | **135.28** | **0.28** |
| **MRPL33** | **135.29** | **0.29** |
| **PPP1R1B** | **135.32** | **0.32** |
| **ATP5I** | **135.47** | **0.47** |
| **SFT2D3** | **135.48** | **0.48** |
| **ARL8B** | **135.51** | **0.51** |
| **CSRP1** | **135.61** | **0.61** |
| **PTPN11** | **135.74** | **0.74** |
| **ZNF146** | **135.94** | **0.94** |
| **SAA1** | **136.03** | **1.03** |
| **FAM63A** | **136.06** | **1.06** |
| **RILPL2** | **136.14** | **1.14** |
| **FAM8A1** | **136.17** | **1.17** |
| **CDH3** | **136.19** | **1.19** |
| **MRPS15** | **136.22** | **1.22** |
| **PDCD4** | **136.40** | **1.40** |
| **KCNMB1** | **136.49** | **1.49** |
| **PDE8A** | **136.57** | **1.57** |
| **RDH10** | **136.58** | **1.58** |
| **MTX2** | **136.69** | **1.69** |
| **MRPS26** | **136.96** | **1.96** |
| **ALDH5A1** | **137.01** | **2.01** |
| **GPX4** | **137.02** | **2.02** |
| **SRXN1** | **137.36** | **2.36** |
| **IGFBP5** | **137.37** | **2.37** |
| **FAM175B** | **137.49** | **2.49** |

**B**

| **Symbol** | **Arc** | **Declination** |
| --- | --- | --- |
| **FKBP2** | **132.55** | **2.45** |
| **LIPH** | **132.56** | **2.44** |
| **TMOD2** | **132.65** | **2.35** |
| **ZC3H11A** | **132.89** | **2.11** |
| **SAA1** | **132.95** | **2.05** |
| **HINT1** | **133.01** | **1.99** |
| **H2AFV** | **133.12** | **1.88** |
| **OGFOD2** | **133.13** | **1.87** |
| **HNRNPA1L2** | **133.29** | **1.71** |
| **OOEP** | **133.30** | **1.70** |
| **PHOX2A** | **133.33** | **1.67** |
| **TOMM20** | **133.37** | **1.63** |
| **RPL7A** | **133.38** | **1.62** |
| **FNBP1** | **133.40** | **1.60** |
| **FAM132A** | **133.47** | **1.53** |
| **TMPRSS4** | **133.47** | **1.53** |
| **PSMB3** | **133.51** | **1.49** |
| **C11orf31** | **133.56** | **1.44** |
| **BST2** | **133.62** | **1.38** |
| **HBP1** | **133.66** | **1.34** |
| **LITAF** | **133.67** | **1.33** |
| **CDC34** | **133.80** | **1.20** |
| **LYN** | **133.92** | **1.08** |
| **FGD6** | **133.93** | **1.07** |
| **PLEKHF1** | **133.94** | **1.06** |
| **ASS1** | **133.99** | **1.01** |
| **STXBP3** | **134.02** | **0.98** |
| **FBXO33** | **134.02** | **0.98** |
| **HDLBP** | **134.04** | **0.96** |
| **SUMF2** | **134.08** | **0.92** |
| **MYL6B** | **134.10** | **0.90** |
| **DNAJA3** | **134.15** | **0.85** |
| **TNFRSF18** | **134.20** | **0.80** |
| **C20orf141** | **134.25** | **0.75** |
| **ERC2** | **134.34** | **0.66** |
| **C17orf75** | **134.48** | **0.52** |
| **MRPL51** | **134.57** | **0.43** |
| **LRP4** | **134.62** | **0.38** |
| **ATP6V1D** | **134.70** | **0.30** |
| **CRK** | **134.70** | **0.30** |
| **ZNF710** | **134.74** | **0.26** |
| **HM13** | **134.79** | **0.21** |
| **FASN** | **134.95** | **0.05** |
| **ARL5A** | **135.00** | **0.00** |
| **FUCA1** | **135.01** | **0.01** |
| **NDUFA7** | **135.02** | **0.02** |
| **CRELD2** | **135.08** | **0.08** |
| **HCFC1R1** | **135.12** | **0.12** |
| **KCTD13** | **135.13** | **0.13** |
| **GPR17** | **135.17** | **0.17** |
| **SEC61B** | **135.29** | **0.29** |
| **SLC37A3** | **135.36** | **0.36** |
| **NUP214** | **135.37** | **0.37** |
| **RPL5** | **135.42** | **0.42** |
| **YPEL3** | **135.51** | **0.51** |
| **HPGD** | **135.52** | **0.52** |
| **NDUFS7** | **135.55** | **0.55** |
| **SFRP1** | **135.56** | **0.56** |
| **BTG3** | **135.59** | **0.59** |
| **TM9SF1** | **135.65** | **0.65** |
| **GNG3** | **135.65** | **0.65** |
| **TRIM7** | **135.71** | **0.71** |
| **CCM2** | **135.81** | **0.81** |
| **SLC39A3** | **135.94** | **0.94** |
| **EXOSC4** | **135.95** | **0.95** |
| **SETD1B** | **135.98** | **0.98** |
| **SLC25A4** | **135.98** | **0.98** |
| **MATK** | **136.13** | **1.13** |
| **YME1L1** | **136.13** | **1.13** |
| **MMADHC** | **136.13** | **1.13** |
| **NUCB2** | **136.19** | **1.19** |
| **ALAS1** | **136.25** | **1.25** |
| **ZZZ3** | **136.25** | **1.25** |
| **ZDBF2** | **136.33** | **1.33** |
| **PFDN5** | **136.52** | **1.52** |
| **COMTD1** | **136.58** | **1.58** |
| **DDX19B** | **136.61** | **1.61** |
| **TM9SF3** | **136.62** | **1.62** |
| **EN1** | **136.62** | **1.62** |
| **DAB2** | **136.68** | **1.68** |
| **SLC35B1** | **136.81** | **1.81** |
| **AVP** | **136.81** | **1.81** |
| **MCL1** | **136.84** | **1.84** |
| **EIF2AK2** | **136.94** | **1.94** |
| **GM2A** | **136.94** | **1.94** |
| **EIF3H** | **136.99** | **1.99** |
| **IL17RD** | **137.04** | **2.04** |
| **DBNDD2** | **137.07** | **2.07** |
| **PRC1** | **137.08** | **2.08** |
| **TRABD** | **137.09** | **2.09** |
| **TM9SF2** | **137.15** | **2.15** |
| **COQ5** | **137.20** | **2.20** |
| **SP8** | **137.20** | **2.20** |
| **TBC1D7** | **137.29** | **2.29** |
| **RNF38** | **137.31** | **2.31** |
| **PPP4R1** | **137.49** | **2.49** |
| **TRIB3** | **137.50** | **2.50** |

**Supplementary Table 6 Identification of genes in the “±2.5°” range from “y = −x.” The angles of polar representation for each gene are shown in the Arc column. The values in the Declination column show each deviation from the angle of −45° or 135° in the polar coordinates. Methylation was detected down-stream of the gene. A. The genes in the 4th quadrant of CSCs treated with 5-FU for 72 h. B. The genes in the 4th quadrant of CSCs treated with CDDP for 72 h.**

**A**

| **Symbol** | **Arc** | **Declination** |
| --- | --- | --- |
| **ARF6** | **−45.24** | **0.24** |
| **DARS** | **−45.01** | **0.01** |
| **FTSJ3** | **−44.58** | **0.42** |
| **MCM5** | **−44.30** | **0.70** |
| **HNRNPC** | **−43.84** | **1.16** |
| **HEATR2** | **−43.50** | **1.50** |
| **GCLM** | **−43.17** | **1.83** |

**B**

| **Symbol** | **Arc** | **Declination** |
| --- | --- | --- |
| **CCND1** | **−46.66** | **1.66** |
| **HEATR2** | **−46.45** | **1.45** |
| **XRCC6** | **−46.35** | **1.35** |
| **MRPL37** | **−46.21** | **1.21** |
| **XPO1** | **−44.95** | **0.05** |
| **C19orf47** | **−44.85** | **0.15** |
| **LRRC59** | **−43.90** | **1.10** |
| **HSPD1** | **−43.77** | **1.23** |
| **CERK** | **−43.73** | **1.27** |
| **B3GNT3** | **−43.43** | **1.57** |
| **RPP30** | **−43.33** | **1.67** |
| **RAN** | **−43.31** | **1.69** |
| **SSNA1** | **−42.81** | **2.19** |

**Supplementary Table 7 Identification of genes in the “±2.5°” range from “y = −x.” The angles of polar representation for each gene are shown in the Arc column. The values in the Declination column show each deviation from the angle of −45° or 135° in the polar coordinates. Methylation was detected down-stream of the gene body. A. The genes in the 4th quadrant of non-CSCs treated with 5-FU for 72 h. B. The genes in the 4th quadrant of non-CSCs treated with CDDP for 72 h.**

**A**

| **Symbol** | **Arc** | **Declination** |
| --- | --- | --- |
| **PCNP** | **−47.29** | **2.29** |
| **SETD2** | **−47.09** | **2.09** |
| **IL13RA1** | **−46.39** | **1.39** |
| **MRPL19** | **−46.36** | **1.36** |
| **NUP35** | **−46.23** | **1.23** |
| **UMPS** | **−45.77** | **0.77** |
| **CDC37** | **−45.53** | **0.53** |
| **MAPKAPK3** | **−45.40** | **0.40** |
| **THAP4** | **−45.40** | **0.40** |
| **PPIF** | **−45.38** | **0.38** |
| **RPL22** | **−45.36** | **0.36** |
| **LYPD2** | **−44.95** | **0.05** |
| **AURKA** | **−44.24** | **0.76** |
| **CTCF** | **−44.07** | **0.93** |
| **SLC25A39** | **−43.99** | **1.01** |
| **RPA1** | **−43.86** | **1.14** |
| **CHAF1A** | **−43.82** | **1.18** |
| **TUFM** | **−43.29** | **1.71** |
| **ZC3HC1** | **−42.70** | **2.30** |
| **THYN1** | **−42.68** | **2.32** |

**B**

| **Symbol** | **Arc** | **Declination** |
| --- | --- | --- |
| **RPA1** | **−47.35** | **2.35** |
| **RFC2** | **−46.13** | **1.13** |
| **FAM111B** | **−45.37** | **0.37** |
| **ALG3** | **−45.16** | **0.16** |
| **HNRNPH3** | **−44.49** | **0.51** |
| **BATF** | **−44.42** | **0.58** |
| **GMNN** | **−43.84** | **1.16** |
| **AK2** | **−43.48** | **1.52** |
| **MRPL4** | **−43.31** | **1.69** |
| **PNN** | **−43.05** | **1.95** |
| **STOML2** | **−42.79** | **2.21** |

**Supplementary Table 8 Identification of the genes in the “±2.5°” range from “y = −x.” The angles of polar representation for each gene are shown in the Arc column. The values in the Declination column show each deviation from the angle of −45° or 135° in the polar coordinates. Methylation was detected within the gene. A. The genes in the 2nd quadrant of CSCs treated with 5-FU for 72 h. B. The genes in the 2nd quadrant of CSCs treated with CDDP for 72 h.**

**A**

| **Symbol** | **Arc** | **Declination** |
| --- | --- | --- |
| **SMARCC1** | **132.50** | **2.50** |
| **POMZP3** | **132.67** | **2.33** |
| **HSDL2** | **132.68** | **2.32** |
| **SIRT3** | **132.79** | **2.21** |
| **BCKDHB** | **132.82** | **2.18** |
| **DMRTC2** | **132.84** | **2.16** |
| **TOM1L1** | **132.87** | **2.13** |
| **TTC8** | **132.87** | **2.13** |
| **SUCLA2** | **132.96** | **2.04** |
| **DERA** | **132.98** | **2.02** |
| **GOLPH3** | **132.98** | **2.02** |
| **ZNF713** | **133.09** | **1.91** |
| **PLD1** | **133.10** | **1.90** |
| **ENOPH1** | **133.12** | **1.88** |
| **SMEK2** | **133.12** | **1.88** |
| **APOA1** | **133.13** | **1.87** |
| **VIPR2** | **133.14** | **1.86** |
| **GTF2E2** | **133.35** | **1.65** |
| **MAT2B** | **133.42** | **1.58** |
| **DNM2** | **133.45** | **1.55** |
| **KCMF1** | **133.47** | **1.53** |
| **MTHFD1** | **133.51** | **1.49** |
| **GRAMD1C** | **133.62** | **1.38** |
| **CASP8** | **133.72** | **1.28** |
| **KLHDC2** | **133.78** | **1.22** |
| **FBXO3** | **133.92** | **1.08** |
| **HSP90B1** | **133.97** | **1.03** |
| **MYO1E** | **134.00** | **1.00** |
| **SYS1** | **134.09** | **0.91** |
| **SUZ12** | **134.11** | **0.89** |
| **FAM171A2** | **134.15** | **0.85** |
| **PXK** | **134.17** | **0.83** |
| **TMEM196** | **134.19** | **0.81** |
| **IL1A** | **134.20** | **0.80** |
| **CORO1B** | **134.21** | **0.79** |
| **MYC** | **134.29** | **0.71** |
| **FAM8A1** | **134.35** | **0.65** |
| **ATXN2L** | **134.45** | **0.55** |
| **MBD5** | **134.45** | **0.55** |
| **ZC3H18** | **134.66** | **0.34** |
| **SPOCK2** | **134.67** | **0.33** |
| **GPC6** | **134.69** | **0.31** |
| **SEMA3A** | **134.75** | **0.25** |
| **ESRP1** | **134.77** | **0.23** |
| **HPS3** | **134.81** | **0.19** |
| **TUBA8** | **134.90** | **0.10** |
| **DYNLT1** | **134.94** | **0.06** |
| **VRK3** | **134.97** | **0.03** |
| **APOA1BP** | **134.98** | **0.02** |
| **TMEM159** | **134.99** | **0.01** |
| **NLRP4** | **135.02** | **0.02** |
| **ATG12** | **135.03** | **0.03** |
| **SETD1A** | **135.06** | **0.06** |
| **LTB4R** | **135.08** | **0.08** |
| **SLCO4A1** | **135.08** | **0.08** |
| **ACSL3** | **135.10** | **0.10** |
| **BIK** | **135.14** | **0.14** |
| **SYT1** | **135.20** | **0.20** |
| **MYO1F** | **135.22** | **0.22** |
| **NCAPH2** | **135.29** | **0.29** |
| **METTL3** | **135.33** | **0.33** |
| **HOOK2** | **135.47** | **0.47** |
| **HINT3** | **135.47** | **0.47** |
| **CDA** | **135.48** | **0.48** |
| **TRAPPC4** | **135.50** | **0.50** |
| **GEM** | **135.50** | **0.50** |
| **C16orf72** | **135.51** | **0.51** |
| **PIAS4** | **135.52** | **0.52** |
| **SEC22B** | **135.56** | **0.56** |
| **PEF1** | **135.67** | **0.67** |
| **GDAP2** | **135.82** | **0.82** |
| **UIMC1** | **135.90** | **0.90** |
| **C4orf33** | **135.94** | **0.94** |
| **NSMCE4A** | **135.94** | **0.94** |
| **STMN3** | **135.99** | **0.99** |
| **SLCO4C1** | **136.07** | **1.07** |
| **ARHGEF19** | **136.12** | **1.12** |
| **GPC2** | **136.13** | **1.13** |
| **PDK2** | **136.16** | **1.16** |
| **TTC28** | **136.17** | **1.17** |
| **DPPA2** | **136.18** | **1.18** |
| **EEA1** | **136.19** | **1.19** |
| **FAM178B** | **136.28** | **1.28** |
| **PDPN** | **136.34** | **1.34** |
| **ABI2** | **136.41** | **1.41** |
| **SNX5** | **136.41** | **1.41** |
| **DVL1** | **136.43** | **1.43** |
| **SNX1** | **136.44** | **1.44** |
| **NEFH** | **136.45** | **1.45** |
| **TRAPPC2L** | **136.52** | **1.52** |
| **KANK2** | **136.56** | **1.56** |
| **RABGAP1** | **136.65** | **1.65** |
| **RIPK4** | **136.68** | **1.68** |
| **NME7** | **136.74** | **1.74** |
| **IFI27L1** | **136.75** | **1.75** |
| **ZC3H4** | **136.78** | **1.78** |
| **INSIG1** | **136.80** | **1.80** |
| **CACNG7** | **136.85** | **1.85** |
| **GNAQ** | **137.03** | **2.03** |
| **ATRNL1** | **137.03** | **2.03** |
| **XPC** | **137.07** | **2.07** |
| **PCDHA7** | **137.08** | **2.08** |
| **EYA4** | **137.11** | **2.11** |
| **USP39** | **137.14** | **2.14** |
| **NCALD** | **137.18** | **2.18** |
| **CYB561D2** | **137.19** | **2.19** |
| **RIPK2** | **137.23** | **2.23** |
| **EIF1AX** | **137.27** | **2.27** |
| **DPYD** | **137.31** | **2.31** |
| **DLL1** | **137.35** | **2.35** |
| **PFDN1** | **137.47** | **2.47** |
| **MED30** | **137.48** | **2.48** |
| **CENPP** | **137.48** | **2.48** |
| **RBM24** | **137.49** | **2.49** |

**B**

| **Symbol** | **Arc** | **Declination** |
| --- | --- | --- |
| **ZFYVE19** | **132.55** | **2.45** |
| **PRKAR2A** | **132.67** | **2.33** |
| **CPNE6** | **132.78** | **2.22** |
| **SERBP1** | **133.14** | **1.86** |
| **HDAC4** | **133.15** | **1.85** |
| **CR1** | **133.24** | **1.76** |
| **KCNMB4** | **133.29** | **1.71** |
| **RGS10** | **133.33** | **1.67** |
| **RTN4RL1** | **133.34** | **1.66** |
| **PON2** | **133.42** | **1.58** |
| **COMMD10** | **133.44** | **1.56** |
| **PRKAA2** | **133.50** | **1.50** |
| **MMADHC** | **133.51** | **1.49** |
| **NIPSNAP1** | **133.64** | **1.36** |
| **CTSH** | **133.71** | **1.29** |
| **MAP4K3** | **133.85** | **1.15** |
| **LSM3** | **133.85** | **1.15** |
| **CDK8** | **133.86** | **1.14** |
| **CEACAM1** | **133.89** | **1.11** |
| **WRB** | **133.93** | **1.07** |
| **CALM1** | **133.95** | **1.05** |
| **GLUD1** | **134.06** | **0.94** |
| **POT1** | **134.11** | **0.89** |
| **TDRD7** | **134.12** | **0.88** |
| **AP3D1** | **134.12** | **0.88** |
| **SGCB** | **134.17** | **0.83** |
| **SAMD8** | **134.25** | **0.75** |
| **TGFBRAP1** | **134.28** | **0.72** |
| **HMGCLL1** | **134.40** | **0.60** |
| **SLC25A4** | **134.40** | **0.60** |
| **P2RX4** | **134.57** | **0.43** |
| **SERPINI1** | **134.60** | **0.40** |
| **DAZAP1** | **134.61** | **0.39** |
| **SNUPN** | **134.72** | **0.28** |
| **E2F5** | **134.74** | **0.26** |
| **IMPAD1** | **134.89** | **0.11** |
| **RRAGD** | **134.93** | **0.07** |
| **KRCC1** | **135.00** | **0.00** |
| **PLEKHF1** | **135.01** | **0.01** |
| **CUL4A** | **135.05** | **0.05** |
| **FASTKD1** | **135.08** | **0.08** |
| **PCDHA2** | **135.13** | **0.13** |
| **TMEM132B** | **135.14** | **0.14** |
| **TERF1** | **135.42** | **0.42** |
| **TMEM159** | **135.44** | **0.44** |
| **TCP10L** | **135.46** | **0.46** |
| **SLCO4A1** | **135.47** | **0.47** |
| **SUSD3** | **135.47** | **0.47** |
| **ABHD14B** | **135.50** | **0.50** |
| **GRTP1** | **135.58** | **0.58** |
| **CEP68** | **135.64** | **0.64** |
| **MED4** | **135.65** | **0.65** |
| **C15orf48** | **135.67** | **0.67** |
| **HRSP12** | **135.73** | **0.73** |
| **CDKN1A** | **135.98** | **0.98** |
| **CKAP4** | **136.11** | **1.11** |
| **MMP28** | **136.18** | **1.18** |
| **ASRGL1** | **136.21** | **1.21** |
| **AFTPH** | **136.22** | **1.22** |
| **RNF125** | **136.24** | **1.24** |
| **ILVBL** | **136.27** | **1.27** |
| **PTPN9** | **136.30** | **1.30** |
| **CPSF3** | **136.36** | **1.36** |
| **IGFBP7** | **136.59** | **1.59** |
| **NUSAP1** | **136.88** | **1.88** |
| **NDUFAF2** | **136.93** | **1.93** |
| **ADAT1** | **137.00** | **2.00** |
| **POLD2** | **137.04** | **2.04** |
| **IVD** | **137.08** | **2.08** |
| **TCTEX1D2** | **137.11** | **2.11** |
| **NDRG4** | **137.13** | **2.13** |
| **KLC2** | **137.13** | **2.13** |
| **INPP4B** | **137.16** | **2.16** |
| **COG4** | **137.29** | **2.29** |
| **GPC2** | **137.32** | **2.32** |
| **KIF3B** | **137.33** | **2.33** |
| **ACSL3** | **137.35** | **2.35** |
| **TRAPPC6B** | **137.37** | **2.37** |
| **RALGPS2** | **137.45** | **2.45** |
| **C9orf78** | **137.49** | **2.49** |

**Supplementary Table 9 Identification of genes in the “±2.5°” range from “y = −x.” The angles of polar representation for each gene are shown in the Arc column. The values in the Declination column show each deviation from the angle of −45° or 135° in the polar coordinates. Methylation was detected within the gene. A. The genes in the 2nd quadrant of non-CSCs treated with 5-FU for 72 h. B. The genes in the 2nd quadrant of non-CSCs treated with CDDP for 72 h.**

**A**

| **Symbol** | **Arc** | **Declination** |
| --- | --- | --- |
| **UBE2H** | **132.51** | **2.49** |
| **MOSPD1** | **132.58** | **2.42** |
| **ABI2** | **132.61** | **2.39** |
| **CTBP2** | **132.63** | **2.37** |
| **OSMR** | **132.67** | **2.33** |
| **PTGES** | **132.68** | **2.32** |
| **SLC35B1** | **132.75** | **2.25** |
| **PPIB** | **132.87** | **2.13** |
| **PKHD1** | **132.90** | **2.10** |
| **TMEM66** | **132.99** | **2.01** |
| **NCAPH2** | **133.09** | **1.91** |
| **DHX15** | **133.13** | **1.87** |
| **RND3** | **133.14** | **1.86** |
| **MPPE1** | **133.16** | **1.84** |
| **MRPL10** | **133.18** | **1.82** |
| **HDLBP** | **133.35** | **1.65** |
| **CPD** | **133.39** | **1.61** |
| **YTHDF1** | **133.42** | **1.58** |
| **PLA2G7** | **133.45** | **1.55** |
| **PMM1** | **133.47** | **1.53** |
| **TUBGCP2** | **133.50** | **1.50** |
| **C15orf61** | **133.50** | **1.50** |
| **HK2** | **133.53** | **1.47** |
| **AP1B1** | **133.62** | **1.38** |
| **CLTA** | **133.62** | **1.38** |
| **ERBB2** | **133.71** | **1.29** |
| **ZMYM2** | **133.82** | **1.18** |
| **TNFAIP1** | **133.85** | **1.15** |
| **CASP9** | **133.90** | **1.10** |
| **NME7** | **133.98** | **1.02** |
| **SNAP23** | **134.02** | **0.98** |
| **PTPN12** | **134.09** | **0.91** |
| **PPP1R14C** | **134.10** | **0.90** |
| **TAL1** | **134.10** | **0.90** |
| **ZNF287** | **134.11** | **0.89** |
| **TNR** | **134.11** | **0.89** |
| **SERINC1** | **134.17** | **0.83** |
| **SUMF2** | **134.21** | **0.79** |
| **PIAS4** | **134.26** | **0.74** |
| **ATP2C1** | **134.26** | **0.74** |
| **PFKP** | **134.30** | **0.70** |
| **PAX6** | **134.35** | **0.65** |
| **TBX5** | **134.42** | **0.58** |
| **RND1** | **134.50** | **0.50** |
| **CTNND1** | **134.54** | **0.46** |
| **TMEM82** | **134.65** | **0.35** |
| **CHID1** | **134.67** | **0.33** |
| **SDF4** | **134.78** | **0.22** |
| **FAM91A1** | **134.79** | **0.21** |
| **CMTM8** | **134.96** | **0.04** |
| **MGEA5** | **135.03** | **0.03** |
| **SPR** | **135.05** | **0.05** |
| **KIN** | **135.12** | **0.12** |
| **AXIN1** | **135.13** | **0.13** |
| **VPS29** | **135.14** | **0.14** |
| **TMEM64** | **135.17** | **0.17** |
| **ALS2CL** | **135.21** | **0.21** |
| **PNRC1** | **135.32** | **0.32** |
| **LHX3** | **135.35** | **0.35** |
| **MYL6B** | **135.39** | **0.39** |
| **NDUFV3** | **135.39** | **0.39** |
| **SCEL** | **135.40** | **0.40** |
| **CIZ1** | **135.41** | **0.41** |
| **SNTA1** | **135.45** | **0.45** |
| **PHYH** | **135.55** | **0.55** |
| **GRAP** | **135.65** | **0.65** |
| **CENPE** | **135.67** | **0.67** |
| **MDM2** | **135.71** | **0.71** |
| **EMD** | **135.73** | **0.73** |
| **GSS** | **135.73** | **0.73** |
| **TIGD3** | **135.81** | **0.81** |
| **MUC13** | **135.89** | **0.89** |
| **ACO2** | **135.91** | **0.91** |
| **MEGF8** | **135.99** | **0.99** |
| **RNF130** | **136.07** | **1.07** |
| **PCSK1N** | **136.09** | **1.09** |
| **SERPINB6** | **136.10** | **1.10** |
| **EFNA3** | **136.14** | **1.14** |
| **CPNE7** | **136.15** | **1.15** |
| **TFCP2L1** | **136.22** | **1.22** |
| **ZG16** | **136.22** | **1.22** |
| **FAM83H** | **136.22** | **1.22** |
| **B9D1** | **136.24** | **1.24** |
| **SNX14** | **136.25** | **1.25** |
| **ZNF766** | **136.25** | **1.25** |
| **MIPOL1** | **136.29** | **1.29** |
| **CRIP1** | **136.32** | **1.32** |
| **SLC12A7** | **136.46** | **1.46** |
| **SERPINB1** | **136.48** | **1.48** |
| **BRI3** | **136.48** | **1.48** |
| **GABRG1** | **136.53** | **1.53** |
| **BDNF** | **136.58** | **1.58** |
| **GNPNAT1** | **136.60** | **1.60** |
| **TUBB2B** | **136.62** | **1.62** |
| **LIMA1** | **136.64** | **1.64** |
| **FNDC3B** | **136.65** | **1.65** |
| **MAT2A** | **136.66** | **1.66** |
| **TCP10L** | **136.76** | **1.76** |
| **MAOA** | **136.87** | **1.87** |
| **SFXN1** | **136.90** | **1.90** |
| **ZFYVE28** | **136.96** | **1.96** |
| **ARL2** | **136.96** | **1.96** |
| **ANG** | **136.97** | **1.97** |
| **NCOR2** | **137.10** | **2.10** |
| **SCD** | **137.11** | **2.11** |
| **PVRL2** | **137.12** | **2.12** |
| **HTR3B** | **137.12** | **2.12** |
| **PLEKHB2** | **137.16** | **2.16** |
| **EIF3F** | **137.17** | **2.17** |
| **SLC6A6** | **137.20** | **2.20** |
| **MAPK6** | **137.22** | **2.22** |
| **TRIP12** | **137.34** | **2.34** |
| **CCNG2** | **137.36** | **2.36** |
| **BCAR1** | **137.50** | **2.50** |

**B**

| **Symbol** | **Arc** | **Declination** |
| --- | --- | --- |
| **JOSD1** | **132.52** | **2.48** |
| **LACTB2** | **132.59** | **2.41** |
| **MRPS28** | **132.62** | **2.38** |
| **MFSD1** | **132.66** | **2.34** |
| **ZDHHC4** | **132.72** | **2.28** |
| **KLHL5** | **132.80** | **2.20** |
| **VPS45** | **132.81** | **2.19** |
| **PPP1R14A** | **132.81** | **2.19** |
| **GLT8D1** | **132.88** | **2.12** |
| **EFNA3** | **132.89** | **2.11** |
| **PTPLAD1** | **132.89** | **2.11** |
| **CNOT8** | **132.89** | **2.11** |
| **RERG** | **133.04** | **1.96** |
| **ALDH7A1** | **133.12** | **1.88** |
| **PKHD1** | **133.21** | **1.79** |
| **CCNO** | **133.24** | **1.76** |
| **CFDP1** | **133.24** | **1.76** |
| **VDAC2** | **133.26** | **1.74** |
| **MYO6** | **133.30** | **1.70** |
| **ATP6V1G1** | **133.31** | **1.69** |
| **RLTPR** | **133.31** | **1.69** |
| **BLOC1S2** | **133.35** | **1.65** |
| **CIB2** | **133.36** | **1.64** |
| **EEF2** | **133.39** | **1.61** |
| **EIF3K** | **133.45** | **1.55** |
| **EGR3** | **133.45** | **1.55** |
| **SFT2D1** | **133.47** | **1.53** |
| **ZNF226** | **133.48** | **1.52** |
| **XK** | **133.53** | **1.47** |
| **ZNF707** | **133.55** | **1.45** |
| **FADS1** | **133.55** | **1.45** |
| **C11orf49** | **133.55** | **1.45** |
| **ASAH1** | **133.56** | **1.44** |
| **FNDC3A** | **133.63** | **1.37** |
| **C19orf53** | **133.64** | **1.36** |
| **ADD1** | **133.66** | **1.34** |
| **PRKDC** | **133.68** | **1.32** |
| **GSDMD** | **133.72** | **1.28** |
| **ZBTB45** | **133.73** | **1.27** |
| **PIK3C3** | **133.78** | **1.22** |
| **MRPL45** | **133.80** | **1.20** |
| **ATP5O** | **133.90** | **1.10** |
| **AACS** | **134.05** | **0.95** |
| **HTATIP2** | **134.16** | **0.84** |
| **KCNK1** | **134.17** | **0.83** |
| **KDELR3** | **134.21** | **0.79** |
| **AVPI1** | **134.24** | **0.76** |
| **PPAP2C** | **134.49** | **0.51** |
| **GALNT12** | **134.49** | **0.51** |
| **C16orf45** | **134.55** | **0.45** |
| **GNB1** | **134.60** | **0.40** |
| **CRISP3** | **134.61** | **0.39** |
| **KLF16** | **134.66** | **0.34** |
| **CDK5RAP3** | **134.67** | **0.33** |
| **PQLC3** | **134.68** | **0.32** |
| **SEC63** | **134.69** | **0.31** |
| **ANKRD50** | **134.78** | **0.22** |
| **TNFAIP8L1** | **134.80** | **0.20** |
| **HIF1A** | **134.84** | **0.16** |
| **NHP2L1** | **134.85** | **0.15** |
| **ISG20** | **134.92** | **0.08** |
| **IAH1** | **134.98** | **0.02** |
| **RABEP2** | **134.99** | **0.01** |
| **U2AF1** | **135.10** | **0.10** |
| **CHRD** | **135.11** | **0.11** |
| **SLC1A7** | **135.15** | **0.15** |
| **CD53** | **135.17** | **0.17** |
| **ARL2** | **135.20** | **0.20** |
| **LCOR** | **135.22** | **0.22** |
| **EXOC6** | **135.29** | **0.29** |
| **MAL2** | **135.29** | **0.29** |
| **HAO2** | **135.33** | **0.33** |
| **CAPN2** | **135.34** | **0.34** |
| **NUP160** | **135.36** | **0.36** |
| **RRAGD** | **135.42** | **0.42** |
| **CRYL1** | **135.49** | **0.49** |
| **MRVI1** | **135.50** | **0.50** |
| **GRAMD1B** | **135.51** | **0.51** |
| **DECR1** | **135.57** | **0.57** |
| **MRPS15** | **135.58** | **0.58** |
| **CANX** | **135.60** | **0.60** |
| **NME3** | **135.62** | **0.62** |
| **PPIL3** | **135.67** | **0.67** |
| **HDAC8** | **135.71** | **0.71** |
| **LDHC** | **135.85** | **0.85** |
| **BBX** | **135.86** | **0.86** |
| **PPM1B** | **135.86** | **0.86** |
| **CGN** | **135.86** | **0.86** |
| **CETN3** | **135.99** | **0.99** |
| **DNAJC18** | **136.02** | **1.02** |
| **TM7SF3** | **136.04** | **1.04** |
| **TAF2** | **136.09** | **1.09** |
| **B9D1** | **136.11** | **1.11** |
| **BMF** | **136.25** | **1.25** |
| **BRD3** | **136.27** | **1.27** |
| **TMBIM6** | **136.28** | **1.28** |
| **ATG4A** | **136.38** | **1.38** |
| **KYNU** | **136.38** | **1.38** |
| **STK16** | **136.45** | **1.45** |
| **IL1RAP** | **136.49** | **1.49** |
| **OXA1L** | **136.51** | **1.51** |
| **CSNK1E** | **136.60** | **1.60** |
| **CHRM2** | **136.60** | **1.60** |
| **GLRX** | **136.61** | **1.61** |
| **CD46** | **136.63** | **1.63** |
| **UBE2E3** | **136.69** | **1.69** |
| **TIMP2** | **136.70** | **1.70** |
| **DGKZ** | **136.82** | **1.82** |
| **KIF3B** | **136.84** | **1.84** |
| **NDUFB2** | **136.86** | **1.86** |
| **POMZP3** | **136.88** | **1.88** |
| **TRMT11** | **136.92** | **1.92** |
| **GNL3L** | **136.94** | **1.94** |
| **ABCB7** | **136.95** | **1.95** |
| **DNAJA3** | **136.96** | **1.96** |
| **PGLS** | **136.98** | **1.98** |
| **HNRNPUL2** | **136.99** | **1.99** |
| **LGALS8** | **137.07** | **2.07** |
| **EIF4EBP1** | **137.15** | **2.15** |
| **PFAS** | **137.19** | **2.19** |
| **ALDH4A1** | **137.20** | **2.20** |
| **YWHAZ** | **137.21** | **2.21** |
| **SMYD3** | **137.24** | **2.24** |
| **UNC93B1** | **137.32** | **2.32** |
| **CAMK1** | **137.37** | **2.37** |
| **PTGES2** | **137.48** | **2.48** |

**Supplementary Table 10 Identification of genes in the “±2.5°” range from “y = −x.” The angles of polar representation for each gene are shown in the Arc column. The values in the Declination column show each deviation from the angle of −45° or 135° in the polar coordinates. Methylation was detected within the gene. A. The genes in the 4th quadrant of CSCs treated with 5-FU for 72 h. B. The genes in the 4th quadrant of CSCs treated with CDDP for 72 h.**

**A**

| **Symbol** | **Arc** | **Declination** |
| --- | --- | --- |
| **PMEPA1** | **−47.15** | **2.15** |
| **SLC20A1** | **−45.96** | **0.96** |
| **XPO1** | **−45.92** | **0.92** |
| **CCDC65** | **−45.32** | **0.32** |
| **F2RL1** | **−44.92** | **0.08** |
| **MTBP** | **−44.42** | **0.58** |
| **MTHFD1L** | **−44.23** | **0.77** |
| **CSK** | **−43.82** | **1.18** |
| **BCR** | **−43.07** | **1.93** |
| **UBE2Z** | **−42.86** | **2.14** |
| **EIF5B** | **−42.59** | **2.41** |

**B**

| **Symbol** | **Arc** | **Declination** |
| --- | --- | --- |
| **HDGF** | **−47.10** | **2.10** |
| **FAM105A** | **−46.82** | **1.82** |
| **RAB7A** | **−46.40** | **1.40** |
| **NEK6** | **−45.56** | **0.56** |
| **RPS6KB2** | **−45.54** | **0.54** |
| **NGDN** | **−44.67** | **0.33** |
| **RBMX** | **−44.44** | **0.56** |
| **C19orf48** | **−44.10** | **0.90** |
| **MCM7** | **−43.72** | **1.28** |
| **SLC41A2** | **−43.41** | **1.59** |
| **SLC20A1** | **−43.28** | **1.72** |
| **UBR7** | **−42.88** | **2.12** |
| **EIF5B** | **−42.65** | **2.35** |

**Supplementary Table11 Identification of genes in the “±2.5°” range from “y = −x.” The angles of polar representation for each gene are shown in the Arc column. The values in the Declination column show each deviation from the angle of −45° or 135° in the polar coordinates. Methylation was detected within the gene. A. The genes in the 4th quadrant of non-CSCs treated with 5-FU for 72 h. B. The genes in the 4th quadrant of non-CSCs treated with CDDP for 72 h.**

**A**

| **Symbol** | **Arc** | **Declination** |
| --- | --- | --- |
| **TAF11** | **−46.34** | **1.34** |
| **KIF11** | **−45.89** | **0.89** |
| **SENP3** | **−45.89** | **0.89** |
| **SERPINH1** | **−45.42** | **0.42** |
| **ECHS1** | **−44.23** | **0.77** |
| **TRMT1** | **−44.15** | **0.85** |

**B**

| **Symbol** | **Arc** | **Declination** |
| --- | --- | --- |
| **ZMAT2** | **−47.09** | **2.09** |
| **EIF5** | **−46.93** | **1.93** |
| **MRTO4** | **−45.68** | **0.68** |
| **EFNA1** | **−43.32** | **1.68** |
| **C5orf24** | **−43.01** | **1.99** |

**Supplementary Table 12 The angles of polar representation of housekeeping genes. The angles of polar representation for each gene are shown in the Arc column. A GAPDH, B**

**A**

|  |  | | **GAPDH** | | | |  |
| --- | --- | --- | --- | --- | --- | --- | --- |
|  |  | | **Methyl** | **Transcriptome** | **M^2^+T^2^** | **Arc** | **Declination** |
| **Zs+** | **5FU** | **UP** | **−0.73** | **0.99** | **1.51** | **126.37** | **8.63** |
|  |  | **Inside** | **0.00** | **0.99** | **0.98** | **90.00** | **45.00** |
|  |  | **Down** | **1.47** | **0.99** | **3.13** | **34.05** | **10.95** |
|  |  | **All** | **0.35** | **0.99** | **1.11** | **70.38** | **25.38** |
|  |  |  |  |  |  |  |  |
|  | **CDDP** | **UP** | **−0.96** | **5.68** | **33.24** | **99.62** | **35.38** |
|  |  | **Inside** | **0.00** | **5.68** | **32.31** | **90.00** | **45.00** |
|  |  | **Down** | **1.15** | **5.68** | **33.64** | **78.53** | **33.53** |
|  |  | **All** | **0.07** | **5.68** | **32.32** | **89.31** | **44.31** |
|  |  |  |  |  |  |  |  |
| **Zs-** | **5FU** | **UP** | **−1.37** | **−1.97** | **5.76** | **−124.84** | **10.16** |
|  |  | **Inside** | **0.00** | **−1.97** | **3.88** | **−90.00** | **45.00** |
|  |  | **Down** | **−0.92** | **−1.97** | **4.72** | **−114.96** | **20.04** |
|  |  | **All** | **−1.23** | **−1.97** | **5.39** | **−121.89** | **13.11** |
|  |  |  |  |  |  |  |  |
|  | **CDDP** | **UP** | **−1.59** | **1.61** | **5.11** | **134.58** | **0.42** |
|  |  | **Inside** | **0.00** | **1.61** | **2.59** | **90.00** | **45.00** |
|  |  | **Down** | **−0.97** | **1.61** | **3.54** | **121.09** | **13.91** |
|  |  | **All** | **−1.32** | **1.61** | **4.33** | **129.30** | **5.70** |

**B**

|  |  | | **β-ACTIN　(ACTB)** | | | |  |
| --- | --- | --- | --- | --- | --- | --- | --- |
|  |  | | **Methyl** | **Transcriptome** | **M^2^+T^2^** | **Arc** | **Declination** |
| **Zs+** | **5FU** | **UP** | **0.11** | **3.18** | **10.13** | **87.98** | **42.98** |
|  |  | **Inside** | **−1.83** | **3.18** | **13.47** | **119.94** | **15.06** |
|  |  | **Down** | **−0.38** | **3.18** | **10.26** | **96.80** | **38.20** |
|  |  | **All** | **−1.39** | **3.18** | **12.03** | **113.55** | **21.45** |
|  |  |  |  |  |  |  |  |
|  | **CDDP** | **UP** | **−0.41** | **7.40** | **54.90** | **93.18** | **41.82** |
|  |  | **Inside** | **−1.73** | **7.40** | **57.74** | **103.20** | **31.80** |
|  |  | **Down** | **−0.44** | **7.40** | **54.92** | **93.39** | **41.61** |
|  |  | **All** | **−1.62** | **7.40** | **57.34** | **102.32** | **32.68** |
|  |  |  |  |  |  |  |  |
| **Zs-** | **5FU** | **UP** | **−0.13** | **6.89** | **47.47** | **91.07** | **43.93** |
|  |  | **Inside** | **−1.19** | **6.89** | **48.87** | **99.80** | **35.20** |
|  |  | **Down** | **0.66** | **6.89** | **47.89** | **84.56** | **39.56** |
|  |  | **All** | **−0.50** | **6.89** | **47.71** | **94.18** | **40.82** |
|  |  |  |  |  |  |  |  |
|  | **CDDP** | **UP** | **−0.34** | **−7.04** | **49.66** | **−92.74** | **42.26** |
|  |  | **Inside** | **−0.91** | **−7.04** | **50.38** | **−97.37** | **37.63** |
|  |  | **Down** | **−0.07** | **−7.04** | **49.55** | **−90.54** | **44.46** |
|  |  | **All** | **−0.83** | **−7.04** | **50.23** | **−96.69** | **38.31** |
